# An Fc variant with two mutations confers prolonged serum half-life and enhanced effector functions on IgG antibodies

**DOI:** 10.1038/s12276-022-00870-5

**Published:** 2022-11-01

**Authors:** Sanghwan Ko, Sora Park, Myung Ho Sohn, Migyeong Jo, Byoung Joon Ko, Jung-Hyun Na, Hojin Yoo, Ae Lee Jeong, Kyungsoo Ha, Ju Rang Woo, Chungsu Lim, Jung Hyu Shin, Dohyun Lee, So-Young Choi, Sang Taek Jung

**Affiliations:** 1 Department of Biomedical Sciences, Graduate School, Korea University, Seongbuk-gu, Seoul, 02707 Republic of Korea; 2 Institute of Human Genetics, Korea University College of Medicine, Seoul, Republic of Korea; 3 New Drug Development Center, Osong Medical Innovation Foundation, 123, Cheongju, Chungcheongbuk-do 28160 Republic of Korea; 4 BK21 Graduate Program, Department of Biomedical Sciences, Korea University College of Medicine, Seoul, Republic of Korea; 5 School of Biopharmaceutical and Medical Sciences, Sungshin Women’s University, Seoul, 02844 Republic of Korea; 6 Department of Pharmaceutical Engineering, Sangji University, Wonju, Gangwon-do 26339 Republic of Korea; 7 Biomedical Research Center, Korea University Anam Hospital, Seoul, Republic of Korea

**Keywords:** Antibody therapy, Molecularly targeted therapy, Drug development

## Abstract

The pH-selective interaction between the immunoglobulin G (IgG) fragment crystallizable region (Fc region) and the neonatal Fc receptor (FcRn) is critical for prolonging the circulating half-lives of IgG molecules through intracellular trafficking and recycling. By using directed evolution, we successfully identified Fc mutations that improve the pH-dependent binding of human FcRn and prolong the serum persistence of a model IgG antibody and an Fc-fusion protein. Strikingly, trastuzumab-PFc29 and aflibercept-PFc29, a model therapeutic IgG antibody and an Fc-fusion protein, respectively, when combined with our engineered Fc (Q311R/M428L), both exhibited significantly higher serum half-lives in human FcRn transgenic mice than their counterparts with wild-type Fc. Moreover, in a cynomolgus monkey model, trastuzumab-PFc29 displayed a superior pharmacokinetic profile to that of both trastuzumab-YTE and trastuzumab-LS, which contain the well-validated serum half-life extension Fcs YTE (M252Y/S254T/T256E) and LS (M428L/N434S), respectively. Furthermore, the introduction of two identified mutations of PFc29 (Q311R/M428L) into the model antibodies enhanced both complement-dependent cytotoxicity and antibody-dependent cell-mediated cytotoxicity activity, which are triggered by the association between IgG Fc and Fc binding ligands and are critical for clearing cancer cells. In addition, the effector functions could be turned off by combining the two mutations of PFc29 with effector function-silencing mutations, but the antibodies maintained their excellent pH-dependent human FcRn binding profile. We expect our Fc variants to be an excellent tool for enhancing the pharmacokinetic profiles and potencies of various therapeutic antibodies and Fc-fusion proteins.

## Drug development: antibodies engineered with superior drug properties

The precise addition of two mutations in the “tail” region of therapeutic antibodies could lead to more potent and long-lived drugs. Professor Sang Taek Jung (Korea University, South Korea) and co-workers generated antibodies with mutations in the “Fc” region, a part of the antibody that interacts with cell surface receptors to mediate immune activation. The researchers identified one particular pair of mutations that improved binding to a receptor involved in protecting antibodies from degradation. Drugs built around this doubly mutated Fc had prolonged half-lives in transgenic mice and monkeys. The mutations also enhanced the ability of antibody drugs to eliminate target cells—a property that, if therapeutically appropriate, could be silenced via additional antibody engineering without affecting the drugs’ extended half-life.

## Introduction

Immunoglobulin G (IgG) antibodies, which are maintained at the second-highest level (7–16 g/L) in human serum (after albumin)^1,2^, are key players in both innate and adaptive immunity^3,4^. In addition, maternal IgGs are transferred to the developing fetal immune system, which is vulnerable to infection by various foreign pathogens, and are crucial in neonatal immunity^5^. IgG can perform its various critical immunological functions at the frontline of the human immune system while being maintained at a constant level in serum because of its interaction with the human neonatal Fc receptor (hFcRn)^6–8^. FcRn, which belongs to the major histocompatibility class I (MHC-I) family, is a heterodimeric complex molecule composed of an α-chain and a β2-microglobulin^9^. It protects IgG molecules from catabolism and recycles them through a distinct pH-dependent interaction with the IgG Fc region that takes the form of association in acidified intracellular endosomes and dissociation in extracellular serum^6–8^. The intracellular trafficking and recycling mechanism has been investigated through various biochemical analyses and spatiotemporal imaging techniques^10–12^.

The A-B loops of the CH2 domain, the E-F loops of the CH2 domain, and the G strand of the CH3 domain in the CH2–CH3 interface of human IgG are the major epitopes that interact with human FcRn^9,13^. In particular, histidine residues (H310, H433, and H435) of the IgG Fc region, which are in protonation and deprotonation equilibrium at a slightly acidic pH, enable the association between FcRn and IgG antibodies under endosomal pH conditions (pH 5.5–6.5) and pH-sensitive dissociation at physiological pH conditions (pH 7.0–7.5) in serum^9,14,15^. An understanding of the interaction between FcRn and IgG at the molecular level elucidates the functions of FcRn and IgG in the human immune system. In addition, it has provided the basis for Fc engineering studies to enhance the efficacy of therapeutic IgG antibodies at reduced dosages and administration frequencies^8,16–18^. Among the engineered Fc variants with improved pH-dependent FcRn interactions, the M252Y/S254T/T256E (YTE) and M428L/N434S (LS) variants have been the most studied, and their clinical utilities for prolonging the circulating half-lives of various therapeutic IgG antibodies have been investigated^16,19^. The YTE variant was introduced to effective prophylactic IgG antibodies, namely, Evusheld^®^ (tixagevimab and cilgavimab), which prevents severe acute respiratory syndrome coronavirus-2 (SARS-CoV-2)^20^, and nirsevimab (MEDI8897), which protects against the respiratory syncytial virus (RSV)^21^. The LS variant was incorporated into Ultomiris^®^ (ravulizumab-cwvz), which is used for the treatment of paroxysmal nocturnal hemoglobinuria (PNH)^22^ and VIR-7831 (sotrovimab), a therapeutic antibody against SARS-CoV-2^23^, which were successfully developed and recently approved by the US FDA^24^.

Other Fc variants that extend the serum half-lives of IgG antibodies have been isolated through rational design or combinatorial library screening; their pharmacokinetic profiles were evaluated in human FcRn transgenic or humanized mice after the Fc variants were introduced into therapeutic antibodies. Those antibody Fc variants extended the circulating half-lives in comparison to antibodies containing the YTE or LS Fc variants discovered in previous studies^25–27^. However, their pharmacokinetic properties have not been evaluated in primate animal models, such as the cynomolgus monkey, which is required for the preclinical prediction of antibody half-life in humans^28–30^, or they showed no extended circulating half-lives upon such evaluation.

In this study, we report a novel Fc variant that can confer improved dissociation capability at pH 7.4 while showing human FcRn binding affinity comparable to that of the previously isolated YTE Fc variant under endosomal acidic pH conditions. Trastuzumab-PFc29, with our discovered Fc variant (PFc29), showed a pharmacokinetic profile in a cynomolgus monkey model that was significantly improved compared to trastuzumab or the trastuzumab-Fc variants (YTE or LS) reported in earlier studies. Furthermore, PFc29 showed enhanced multiple functions, indicating high therapeutic potential with the ability to elicit improved effector functions for target tumor cell clearance through enhanced human FcγRs (hFcγRs) and human C1q (hC1q) binding. Additionally, PFc29 blocked effector functions upon the introduction of additional mutations that ablate Fc–hFcγR interactions. Our results indicate that an engineered Fc variant with improved pH-dependent FcRn binding and tunable effector functions could be used to develop therapeutic IgG antibodies and Fc-fusion proteins with prolonged serum persistence and enhanced efficacy.

## Materials and methods

### Reagents

All plasmids and primers used in this study are summarized in Supplementary Tables 1 and 2, respectively. All restriction endonucleases, T4 DNA ligase, and Vent polymerase were purchased from New England Biolabs (Ipswich, MA, USA). The *Taq* polymerase and oligonucleotide primers were from Biosesang (Seongnam, Republic of Korea) and Cosmogenetech (Seoul, Republic of Korea), respectively. Difco^TM^ Terrific Broth, Ni-NTA agarose, Protein A agarose, and glutathione agarose 4B were obtained from Becton Dickinson Diagnostic Systems (Sparks, MD, USA), Qiagen (Hilden, Germany), Genscript (Scotch Plains, NJ, USA), and Incospharm (Daejeon, Republic of Korea), respectively. An Alexa Fluor 488 labeling kit, 1-Step Ultra-TMB substrate solution, GIBCO FreeStyle™ 293 expression medium, and sheep anti-hC1q-HRP conjugate were purchased from Thermo Fisher Scientific (Waltham, MA, USA). Polyethyleneimine-Max and goat anti-GST-HRP conjugate were obtained from Polysciences (Taipei, Taiwan) and GE Healthcare (Piscataway, NJ, USA), respectively. Unless stated otherwise, all other biochemical reagents were purchased from Sigma‒Aldrich (St. Louis, MO, USA).

### Surface plasmon resonance analysis

The affinities of trastuzumab-Fc variants for hFcRn at pH 6.0 were measured by surface plasmon resonance (SPR) on a Biacore T200 instrument. Human epidermal growth factor receptor 2 (HER2) (Sino Biological, Beijing, China) was immobilized on a Series S CM5 sensor chip (GE Healthcare) using amine coupling chemistry to reach ~3000 response units (RU). After capturing trastuzumab-Fc variants (RU = ~300) on the HER2-coated sensor chip, hFcRn, which was serially diluted in 10× phosphate-buffered saline (PBS) and 0.05% Tween 20 at pH 6.0 (1000, 500, 250, 125, and 62.5 nM), was injected over the chip at a flow rate of 50 μl/min for 120 s, followed by an injection of buffer alone for 100 s. Signal detection was performed at a rate of ten signals per second. Binding constants were determined using the 1:1 binding model in Biacore T200 Evaluation software version 3.1 (GE Healthcare). To analyze the RU binding of the trastuzumab-Fc variants to hFcRn at pH 7.4, an anti-His antibody included in a His Capture Kit (Cytiva, Piscataway, NJ, USA) was coated onto a Series S CM5 sensor chip using covalent amine coupling chemistry (RU = 12,000–15,000). After immobilizing monomeric hFcRn by binding it to the anti-His Ab-coated sensor chip (RU = ~170), the surfaces were blocked with an injection of 1 M ethanolamine-HCl (pH 8.5). Antibody Fc variants serially diluted in 1× PBS, 0.05% Tween 20, and 350 mM NaCl at pH 7.4 were injected over the chip at a flow rate of 50 μl/min for 360 s, and then buffer only was injected for 120 s at the same flow rate. For a quantitative binding analysis of the antibody Fc variants to FcγRIIIa-158V, an anti-His antibody was immobilized using the method just described, and FcγRIIIa-158V was injected for 30 s using a flow rate of 5 μl/min. Rituximab-Fc variants serially diluted in PBS, 0.05% Tween 20, and 350 mM NaCl buffer were injected over the chip with an association time of 1 min and a dissociation time of 3 min at a flow rate of 30 μl/min. After each binding cycle, a regeneration solution (10 mM glycine, pH 1.5) was injected for 30 s at a flow rate of 30 μl/min.

### Pharmacokinetic profile analyses

For the pharmacokinetic profiles, hFcRn transgenic mice (B6.Cg-*Fcgrt*^tm1Dcr^ Tg(CAG-FCGRT)276Dcr/DcrJ homozygous) were injected intravenously with 5 mg/kg of the trastuzumab-Fc variants (trastuzumab, trastuzumab-PFc29, or trastuzumab-PFc41) or 4 mg/kg of the aflibercept-Fc variants (aflibercept, aflibercept-PFc29, or aflibercept-PFc41) (*n* = 5). Blood samples were drawn from each mouse via the facial vein at 0.5, 1, 3, 6, 12, 24, 72, 168, 336, 504, 672, 840, 1008, and 1200 h post-injection for the trastuzumab-Fc variants and 0.48, 0.96, 3, 6, 12, 24, 72, 168, and 336 h post-injection for the aflibercept-Fc variants. Cynomolgus monkeys were injected intravenously with 6 mg/kg of trastuzumab-Fc variants (trastuzumab, trastuzumab-YTE, trastuzumab-LS, and trastuzumab-PFc29) (*n* = 2 per variant), and blood samples were drawn via the femoral vein at each time point (0.5, 1, 3, 6, 12, 24, 72, 120, 168, 504, 840, 1176, and 1512 h post-injection). To measure the concentrations of the trastuzumab- and aflibercept-Fc variants in serum samples, MaxiSorp^TM^ flat-bottom 96-well microplates (Thermo Fisher Scientific) were coated with 1 μg/ml HER2 diluted in ELISA Coating Buffer (Bethyl Laboratories, Montgomery, TX, USA) by overnight incubation at 4 °C. After washing the microplates four times with ELISA Wash Solution (Bethyl Laboratories) and adding ELISA Blocking Buffer (Bethyl Laboratories), we incubated the plates at 25 °C for 2 h. After four cycles of washing, standards (purified and quantified trastuzumab-Fc variants) and serum samples drawn from animals were diluted in sample/conjugate diluent (ELISA blocking buffer containing 0.05% Tween 20; Bethyl Laboratories) and added to the plate. After 1.5 h of incubation at 25 °C, four cycles of washing, and the addition of 50 μl of horseradish peroxidase-conjugated goat anti-human IgG Fc diluted 1:20,000-fold in sample/conjugate diluent, the plates were incubated again at room temperature for 1 h and washed four times. The ELISA signal was developed using 1-Step Ultra-TMB substrate, and color development was stopped by adding 50 μl of ELISA Stop Solution (Bethyl Laboratories). After the absorbance was measured at 450 nm on a FilterMax F5 Multi-Mode Microplate Reader (Molecular Devices, San Jose, CA, USA), the concentrations of the samples were determined by extrapolation from a standard curve made in a four-parameter nonlinear regression program in Softmax Pro software (Molecular Devices).

### Antibody-dependent cellular cytotoxicity (ADCC) assays

The antibody-dependent cellular cytotoxicity (ADCC) activity of the trastuzumab-Fc variants was evaluated using an ADCC Reporter Bioassay kit (Promega, Madison, WI, USA). A 96-well plate containing 25 μl of SKBR-3 cells (1.25 × 10^4^ cells per well) and 25 μl of trastuzumab-Fc variants serially diluted at 1 μg/ml was loaded with effector cells, which were prepared according to the manufacturer’s instructions (25 μl/well) (E:T ratio = 6:1). After incubation in a CO_2_ incubator at 37 °C for 6 h, the addition of 75 μl of Bio-Glo^TM^ Luciferase Assay Reagent (Promega), and further incubation at 25 °C for 5 min, the luminescence of each well was measured using an Enspire ELISA plate reader (PerkinElmer, Waltham, MA, USA). The fold-change in the induction of cell lysis for each trastuzumab-Fc variant was calculated using the formula below.

$${{{\mathrm{Fold}}}} - {{{\mathrm{change}}}}\;{{{\mathrm{of}}}}\;{{{\mathrm{induction}}}} = \frac{{{{{\mathrm{Experimental}}}}\;{{{\mathrm{cell}}}}\;{{{\mathrm{death}}}}}}{{{{{\mathrm{Spontaneous}}}}\;{{{\mathrm{target}}}}\;{{{\mathrm{cell}}}}\;{{{\mathrm{death}}}}}}$$

### Complement-dependent cytotoxicity (CDC) assays

The complement-induced lysis of CD20^+^ Raji cells was measured using a normal human complement (Quidel, San Diego, CA, USA) and a CytoTox 96® Non-Radioactive Cytotoxicity Assay kit (Promega). After adding 50 µl of Raji cells (1 × 10^4^ cells) to each well of a sterile, clear, 96-well, V-bottom plate (Corning), 10 µl of rituximab-Fc variants, threefold serially diluted from 1 μg/ml, was added to each well. Then, 40 µl of human serum complement (final concentration of complement = 2.5%) was added to the wells. After centrifugation of the plate at 300 × *g* for 3 min, 50 µl of supernatant from each well was transferred into a Spectraplate 96-well plate (PerkinElmer), and 50 µl of Cytotox 96® reagent (Promega) was added. The plate was further incubated at 25 °C for 30 min. Then, 50 µl of Stop Solution (Promega) was added, and the cytotoxicity was evaluated by measuring the absorbance at 490 nm. The experiments were repeated three times in duplicate, and the average percentage of cytotoxicity was calculated. For the positive control of target cell cytotoxicity (maximum target cell death), 10 μl of 0.9% Triton X-100 in 1 × PBS was added. For the negative control of target cell cytotoxicity (spontaneous target cell death), a sample of target cells that was not treated with an antibody or complement was used. The percentage of cell lysis for each rituximab-Fc variant was calculated using the formula below.

$${{{\mathrm{CDC}}}}\;{{{\mathrm{cytotoxicity}}}}\;\left( \% \right) = {{{\mathrm{100}}}}\;{{{\mathrm{x}}}}\frac{{{{{\mathrm{(Experimental}}}}\;{{{\mathrm{cell}}}}\;{{{\mathrm{death}}}} - {{{\mathrm{Spontaneous}}}}\;{{{\mathrm{target}}}}\;{{{\mathrm{cell}}}}\;{{{\mathrm{death}}}} - {{{\mathrm{Complement}}}}\;{{{\mathrm{absorbance)}}}}}}{{{{{\mathrm{(Maximum}}}}\;{{{\mathrm{target}}}}\;{{{\mathrm{cell}}}}\;{{{\mathrm{death}}}} - {{{\mathrm{Spontaneous}}}}\;{{{\mathrm{target}}}}\;{{{\mathrm{cell}}}}\;{{{\mathrm{death)}}}}}}$$

## Results

### High-throughput directed evolution of Fc for improved pH-dependent human FcRn binding

**Fig. 1:**
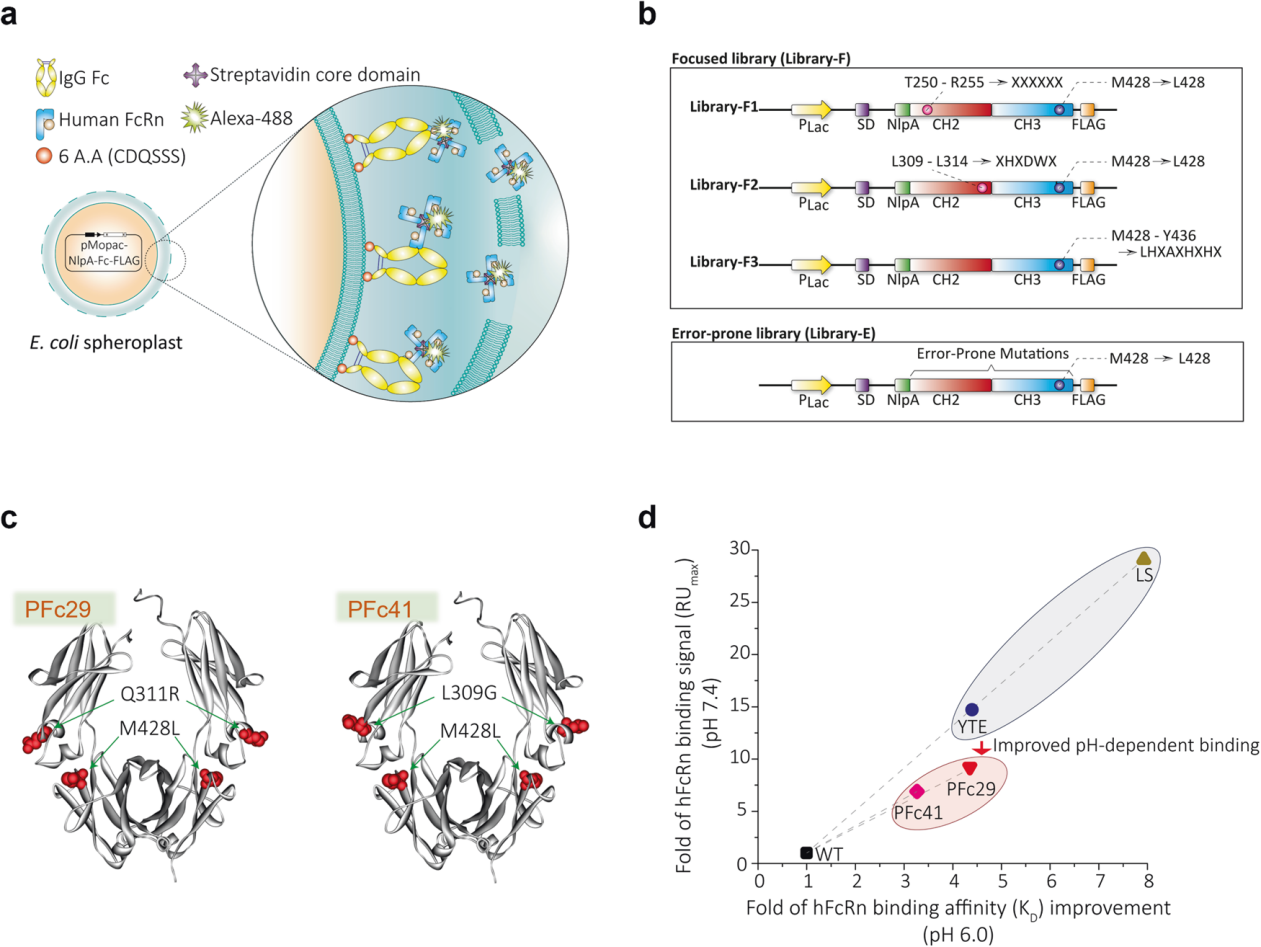
Isolation of Fc variants with improved FcRn binding. **a** Screening strategy for isolating Fc variants using the bacterial display. **b** Schematic diagram displaying the expression cassettes of the mutagenized Fc libraries (Library-F and Library-E). **c** Solid ribbon structure showing the mutation sites for the isolated Fc variants. The mutations identified for PFc29 (left) and PFc41 (right) are overlaid on the structure of the Fc region of the human IgG crystal structure (PDB: 1HZH). **d** The dot plot shows the improved hFcRn binding of the Fc variants relative to a wild-type Fc at pH 6.0 and pH 7.4. The x-axis and y-axis of the dot plot indicate the fold improvement of hFcRn binding affinity (*K*_D_) for the Fc variants at pH 6.0 and the fold improvement of hFcRn binding signal (RU_max_) for the Fc variants at pH 7.4 relative to a wild-type Fc, respectively. The Fc variants and wild-type Fc were introduced into trastuzumab, and *K*_D_ and RU_max_ were measured using an SPR analysis.

hFcRn binds to the Fc region of pinocytosed human serum IgG in acidified endosomes^31^. For efficient screening of Fc variants exhibiting enhanced hFcRn binding profiles, we used a membrane protein drift and assembly (MPDA) bacterial display system^32^, which enabled us to anchor monomeric polypeptides on the periplasmic side of the *Escherichia coli* inner membrane for the autoassembly of polypeptides on a fluidic membrane for the multimeric formation of complex proteins. In our previous work, we found that this MPDA system^32^ showed higher efficiency in displaying and screening homodimeric Fc variants than two previously reported bacterial display systems^33,34^. Considering the low-affinity interaction between native hFcRn and the wild-type Fc domain of human IgG^35^, we used dimeric or tetrameric forms of extracellular hFcRn C-terminally fused to glutathione-S-transferase (GST) or streptavidin to enhance the apparent affinity by increasing the avidity. After Alexa 488 labeling for FcRn-GST or FcRn-streptavidin, these fluorescent probes were incubated with wild-type Fc and Fc variants displayed on the bacterial inner membrane using the MPDA system (Fig. 1a).

To confirm whether that display system can be used to screen Fc variants for improved FcRn binding characteristics, we conducted a flow cytometric analysis using two Fc variants with increased FcRn binding (LS and N434W). As expected, the LS variant, which was reported to enhance hFcRn binding and prolong the circulating half-lives of IgG antibodies^16^, showed a higher hFcRn binding signal than the wild-type Fc. In particular, the N434W variant, which displayed increased hFcRn binding at neutral pH as well as acidic pH^36^, showed increased fluorescence-activated cell sorting (FACS) signals under both pH conditions (Supplementary Fig. 1), suggesting that our Fc display system could be used to screen for Fc variants exhibiting improved hFcRn binding.

In our previous study, we found that the M428L mutation enhanced the hFcRn binding of the Fc domain at pH 6^33^, and the M428L Fc variant displayed on the *E. coli* inner membrane showed a slightly increased hFcRn binding signal in the FACS analysis, as expected (Supplementary Fig. 1). To isolate Fc variants with circulating half-lives prolonged by improved hFcRn binding affinity under endosomal pH conditions, we used the Fc variant containing the M428L substitution mutation as a parental template and constructed two libraries: (i) an error-prone PCR library (Library-E) of random mutations across the entire Fc region and (ii) a focused library (Library-F) containing random mutations in the hFcRn binding site (T250-R255, L309-L314, and M428-Y436) (Fig. 1b). Using the bacterial display system and flow cytometric screening of a mixture of the two libraries (library size >2.2 × 10^8^) (Fig. 1b), we enriched *E. coli* spheroplasts displaying Fc variants with high hFcRn-binding FACS signals at pH 5.8. Then, the hFcRn-binding FACS signals of 40 randomly chosen individual clones rescued from the fourth-round screening were analyzed, and the top four Fc variants (PFc41, EFc29, PFc29, and EFc41) exhibited the highest mean fluorescence intensities (MFIs) (MFI_wild-type Fc_ = 303.75, MFI_PFc41_ = 1913.80, MFI_EFc29_ = 1669.45, MFI_PFc29_ = 1184.35, MFI_EFc41_ = 861.62). Among those Fc variants, PFc29 (Q311R/M428L) and PFc41 (L309G/M428L) had only two substitution mutations in the upper CH3 region corresponding to the putative hFcRn binding site (Fig. 1c and Supplementary Figs. 2, 3a, b). On the other hand, EFc29 and EFc41 had five (P228L/R292L/T359A/S364G/M428L) and seven mutations (P228L/L234F/E269D/Q342L/E388D/T394A/M428L), respectively, some of which were located very far from the hFcRn binding site (Fig. 1c and Supplementary Figs. 2, 3a, b).

### Trastuzumab containing an engineered Fc variant exhibited more pH-sensitive hFcRn binding than trastuzumab-YTE

To characterize the identified Fc variants in a full-length IgG1 format, we introduced the isolated Fc variants into trastuzumab (Herceptin^Ⓡ^) and expressed the antibody Fc variants in Expi293F cells. After we purified the trastuzumab and trastuzumab-Fc variants using Protein A affinity chromatography, we analyzed their aggregation propensities using size exclusion chromatography high-performance liquid chromatography (SEC-HPLC). Although trastuzumab-EFc29 and trastuzumab-EFc41 showed a small high-molecular-weight peak representing an IgG multimer in the SEC-HPLC analysis, trastuzumab-PFc29 and trastuzumab-PFc41 showed a single peak with a retention time identical to that of clinical-grade Herceptin, suggesting that neither PFc29 nor PFc41 increased the aggregation propensity of trastuzumab (Supplementary Fig. 4a–h). Based on those SEC-HPLC results, the two Fc variants (PFc29 and PFc41) exhibiting elution profiles almost identical to clinical-grade trastuzumab (without a small multimeric shoulder peak) were selected for further analyses.

Quantitative affinity constants for the binding between hFcRn and trastuzumab and four trastuzumab-Fc variants (trastuzumab-YTE, trastuzumab-LS, trastuzumab-PFc29, trastuzumab-PFc41) to hFcRn at pH 6.0 were analyzed by SPR. These measurements revealed that two of the trastuzumab-Fc variants (trastuzumab-PFc29 and trastuzumab-PFc41) showed significantly improved affinity for hFcRn at pH 6.0 compared to trastuzumab containing wild-type Fc. In particular, trastuzumab-PFc29 showed an equilibrium dissociation constant almost identical to that of trastuzumab-YTE in binding to hFcRn at pH 6.0 (Fig. 1d and Supplementary Table 3). On the other hand, because the affinities between the trastuzumab-Fc variants and hFcRn at neutral pH (pH 7.4) were not high enough to be analyzed quantitatively, we analyzed the maximum response unit (RU_max_) values upon binding between the trastuzumab-Fc variants and hFcRn immobilized on the SPR sensor chip. In the SPR analysis, trastuzumab-PFc29 and trastuzumab-PFc41 showed lower hFcRn binding at pH 7.4 than trastuzumab-YTE and trastuzumab-LS, which might enable them to more efficiently release IgG antibodies from the late endosome into the serum (Fig. 1d and Supplementary Table 3).

### Two mutations of PFc29 or PFc41 prolong the circulating half-lives of trastuzumab and aflibercept in hFcRn transgenic mice

**Fig. 2:**
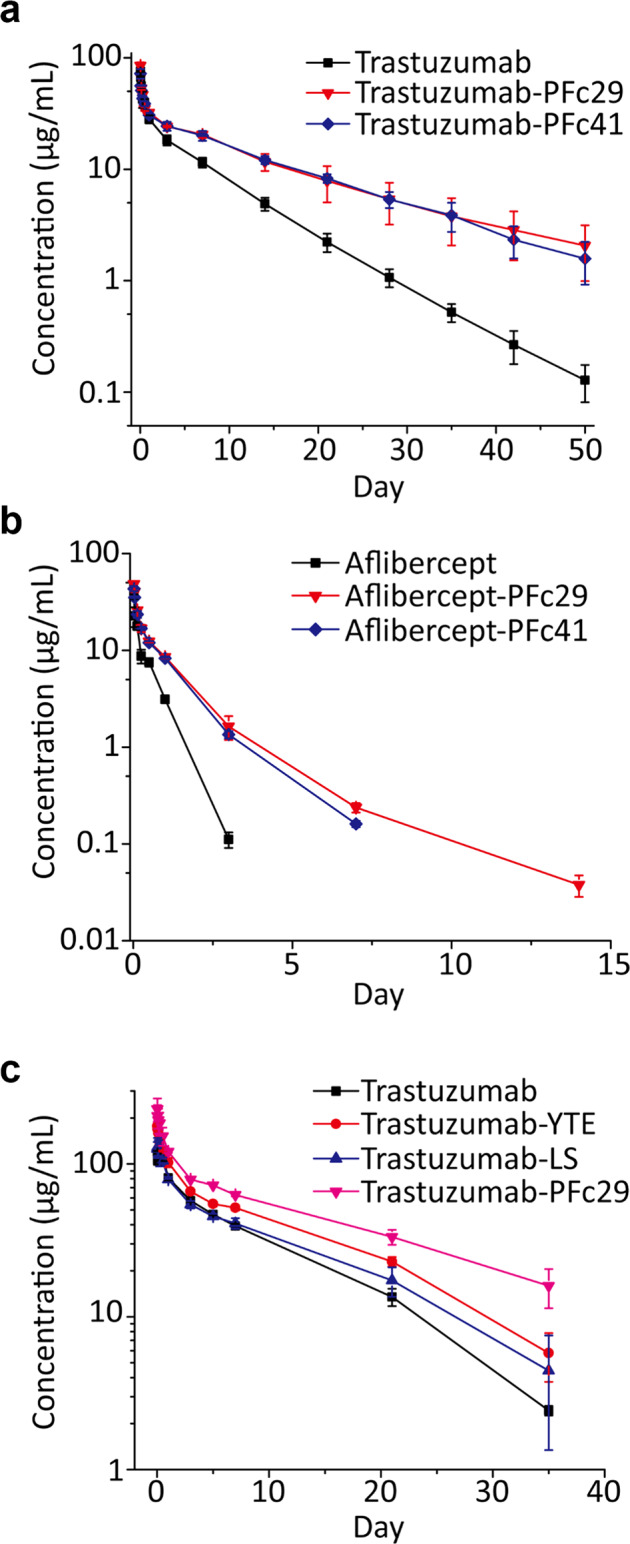
Pharmacokinetic profile analysis of trastuzumab-Fc variants and aflibercept-Fc variants. Serum concentration vs. time profiles for the trastuzumab-Fc variants (5 mg/kg, *n* = 5) **a** and aflibercept-Fc variants (4 mg/kg, *n* = 5) **b** in hFcRn transgenic mice and trastuzumab-Fc variants (6 mg/kg, *n* = 2) **c** in cynomolgus monkeys. Data were presented as the mean values and standard errors.

Considering the higher binding preference of a wild-type human Fc for mFcRn over hFcRn and the discrepancy between the affinity of engineered Fc for human FcRn and mouse FcRn, it is important that the pharmacokinetic profiles of IgGs or Fc-fusion proteins with Fc variants be evaluated in an hFcRn transgenic mouse model instead of normal mice^37^. Therefore, we examined whether the improved pH-dependent hFcRn binding of the identified Fc variants (PFc29 and PFc41) can prolong the in vivo circulating half-lives of IgG antibodies and Fc-fusion proteins in hFcRn transgenic (Tg) mice. Trastuzumab and the trastuzumab-Fc variants were injected into the tail veins (5 mg/kg) of hFcRn Tg mice, and blood was drawn at intervals for 50 days. In the pharmacokinetic profile analysis, both trastuzumab-PFc29 and trastuzumab-PFc41 showed significantly improved serum half-lives and total drug exposure over time (AUC_inf_) compared to trastuzumab, and trastuzumab-PFc29 showed the highest half-life and AUC_inf_ (Fig. 2a and Table 1).

**Table 1:** Noncompartmental pharmacokinetic parameters for hFcRn mouse and monkey studies.

Species^a^	Parameter	t_1/2_ (day)	C_max_ (μg mL^−1^)	AUC_inf_ (μg mL^−1^ day)	AUC_%Extrap_	CL (mL day^−1^)
hFcRn transgenic mouse^1^	Trastuzumab	7.03 ± 1.16	71.14 ± 3.95	247.08 ± 18.60	0.6 ± 0.25	0.41 ± 0.03
	Trastuzumab-PFc29	14.07 ± 2.70	79.30 ± 9.48	528.82 ± 101.78	7.7 ± 3.9	0.21 ± 0.04
	Trastuzumab-PFc41	10.90 ± 2.54	67.91 ± 7.60	480.90 ± 60.65	7.0 ± 0.5	0.22 ± 0.02
hFcRn transgenic mouse^2^	Aflibercept	0.41 ± 0.02	30.55 ± 8.50	15.63 ± 4.06	0.41 ± 0.11	6.40 ± 0.13
	Aflibercept-PFc29	2.10 ± 0.63	45.26 ± 4.42	33.66 ± 4.56	0.40 ± 0.35	2.97 ± 0.09
	Aflibercept-PFc41	1.01 ± 0.03	39.95 ± 5.32	30.33 ± 2.33	0.78 ± 0.14	3.30 ± 1.18
Cyno	Trastuzumab	7.17 ± 0.14	120.38 ± 1.29	881.49 ± 35.68	2.879 ± 0.38	13.64 ± 0.55
	Trastuzumab-YTE	8.84 ± 2.81	130.56 ± 17.43	1001.44 ± 154.20	6.17 ± 4.79	12.27 ± 1.89
	Trastuzumab-LS	8.88 ± 1.17	177.20 ± 10.32	1263.26 ± 42.53	6.05 ± 2.63	9.51 ± 0.32
	Trastuzumab-PFc29	14.34 ± 2.75	228.52 ± 39.46	1966.32 ± 286.39	16.89 ± 5.60	6.24 ± 0.91

*t*_*1/2*_ terminal half-life (β-phase), *C*_max_ maximal concentration, *AUC*_inf_ area under the curve from administration to infinity, *AUC%*_Extrap_ percentage of the extrapolated area under the curve to the total area under the curve, *CL* clearance.

^a^hFcRn transgenic mouse and Cyno refer to Tg276 mouse (B6.Cg-*Fcgrt*^tm1Dcr^ Tg(CAG-FCGRT)276Dcr/DcrJ homozygous) and in cynomolgus monkey, respectively. Dose levels and routes: single i.v. bolus at 5 mg/kg (*n* = 5) for hFcRn transgenic mouse^1^; single i.v. bolus at 4 mg/kg (*n* = 5) for hFcRn transgenic mouse^2^; single i.v. infusion at 6 mg/kg (*n* = 2) for Cyno.

Next, to examine whether the identified Fc variants (PFc29 and PFc41) would improve the serum half-lives of Fc-fusion proteins, we introduced them into a model Fc-fusion protein, aflibercept, in which the Fc region was fused to the vascular endothelial growth factor receptor (VEGFR). After we produced aflibercept and the aflibercept-Fc variants in Expi293F cells, we analyzed their purity using SEC-HPLC, and no detectable aggregation peak was observed (Supplementary Fig. 4i–l). Then, the prepared samples were injected into hFcRn Tg mice (4 mg/kg), and their pharmacokinetic profiles were evaluated. The results clearly demonstrated that both aflibercept-Fc variants (PFc29 and PFc41) had dramatically greater serum persistence than aflibercept. In particular, aflibercept-PFc29 was detected in serum even after 14 days (0.0378 μg/ml), whereas aflibercept and aflibercept-PFc41 were undetectable after 7 days (Fig. 2b and Table 1).

### Trastuzumab-PFc29 displayed an improved pharmacokinetic profile compared with trastuzumab-YTE and trastuzumab-LS in cynomolgus monkeys

In the pharmacokinetic profile analyses of the trastuzumab- and aflibercept-Fc variants in hFcRn Tg mice, the PFc29 variant, which had a higher binding affinity at pH 6.0 than the PFc41 variant, exhibited a more prolonged half-life than the PFc41 variant (Fig. 1d and Table 1). Therefore, we selected the PFc29 variant for further physicochemical characterization and pharmacokinetic analysis in cynomolgus monkeys.

The hFcRn Tg mouse model has been used in many studies for its utility in evaluating the pharmacokinetic profiles of antibodies through hFcRn binding-mediated recycling. However, human FcRn knock-in transgenic mice have lower serum IgG concentrations than normal mice expressing mouse FcRn due to the low binding affinity between human FcRn and mouse IgG. Therefore, the hFcRn Tg mouse models (Tg32 and Tg276) are not suitable for predicting the pharmacokinetic profiles of therapeutic IgG antibodies that exhibit competitive binding to serum IgGs^27^. In contrast, primate animal models have FcRn tissue distributions^38^ and endogenous serum IgG concentrations (8–19 g/L)^39^ similar to those in humans, so primate animals provide more accurate models for predicting the pharmacokinetic profiles of a therapeutic antibody administered to humans^28–30^. Thus, to analyze the pharmacokinetic profiles of the Fc variants (trastuzumab, trastuzumab-YTE, trastuzumab-LS, and trastuzumab-PFc29) in a cynomolgus monkey model, 6 mg/kg of each trastuzumab-Fc variant was administered to cynomolgus monkeys via the cephalic vein, and serum samples were collected and analyzed for 35 days. The results clearly showed that trastuzumab-PFc29 exhibited a significantly prolonged serum half-life (100, 62.2, and 61.5% higher than trastuzumab, trastuzumab-YTE, and trastuzumab-LS, respectively) and AUC_inf_ (123.1, 96.3, and 55.7% higher values than those of trastuzumab, trastuzumab-YTE, and trastuzumab-LS, respectively; Fig. 2c and Table 1). Moreover, trastuzumab-PFc29 had the lowest clearance rate among the three trastuzumab-Fc variants (118.6, 96.6, and 52.4% lower than those of trastuzumab-YTE, trastuzumab-LS, and trastuzumab, respectively; Fig. 2c and Table 1).

### Structural interpretation via complex modeling analysis elucidated the enhanced hFcRn binding of PFc29 at pH 6.0

**Fig. 3:**
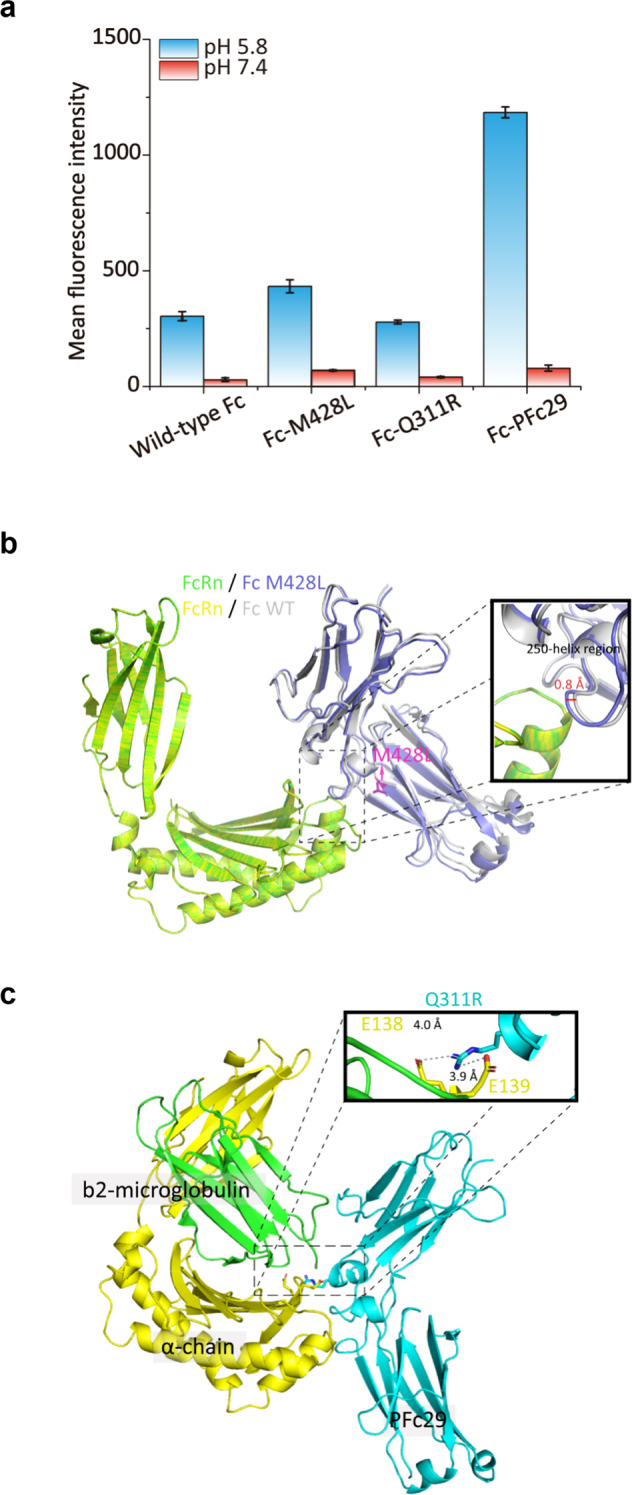
Molecular interpretation of the improved PFc29 binding to hFcRn at pH 6.0. **a** The combinatorial effects of the two mutations (Q311R and M428L) of PFc29 on hFcRn binding. For the FACS analysis, *E. coli* spheroplasts displaying an Fc variant (wild-type Fc, Fc-428L, Fc-Q311R, or Fc-PFc29) were labeled with 40 nM hFcRn-SA-Alexa 488 at pH 6.0 and pH 7.4. The mean fluorescence intensities (MFIs) resulting from the FACS analysis are represented as a bar graph. Error bars indicate the standard deviations calculated from triplicate samples. **b** Superposition of two complex model structures, hFcRn (green)/Fc-M428L (blue) and hFcRn (yellow)/wild-type Fc (white). To examine how the M428L mutation affects the distance between the Fc region and hFcRn, the two complex models (hFcRn/Fc-M428L and hFcRn/wild-type Fc) are superposed based on hFcRn C^α^, and a zoomed-in view showing the interaction between hFcRn and the 250-helix region (residues K246–M252) is outlined with a thick line box. The bidirectional arrow in the box indicates the structural distance between Fc-M428L (blue) and wild-type Fc (gray), and the side chain of the M428L residue of Fc-428L is shown in stick representation (purple). **c** Molecular complex model of PFc29/hFcRn. PFc29, hFcRn α-chain, and β2-microglobulin are colored cyan, yellow, and green, respectively. A contact area close to Q311R is indicated by a box, and electrostatic interactions (Q311R_PFc29_–E138_hFcRn_ or Q311R_PFc29_–E139_hFcRn_) are represented by dashed lines.

To understand why the two mutations of PFc29 (Q311R/M428L) elicited enhanced binding to hFcRn at pH 6.0, we first conducted a back-mutation experiment. The hFcRn binding affinities of Fc variants (Fc-Q311R and Fc-M428L) that contained a single-substitution mutation (Q311R or M428L) were greatly decreased compared with that of PFc29 (Fig. 3a). In particular, the FcRn binding affinity of Fc-Q311R was similar to that of wild-type Fc (Fig. 3a), suggesting that the improved affinity of PFc29 to FcRn at pH 6.0 was affected by the combination of the two substitution mutations (M428L and Q311R).

Although it was reported that the M428L mutation played a crucial role in the enhanced hFcRn interaction with IgG Fc variants to improve the serum half-life^16,33,40^, the molecular mechanism for the interaction between hFcRn and Fc variants containing the M428L mutation remains elusive. To understand the improved hFcRn binding of the M428L mutation, we generated in silico complex models of hFcRn–Fc variants. First, we compared the complex model structures of the hFcRn–Fc-M428L variant and the hFcRn–wild-type Fc. Interestingly, the 250-helix region (amino acid residues: K246–M252)^25^ was significantly changed (0.8 Å) by the M428L substitution mutation alone (Fig. 3b), suggesting that the conformational change caused by M428L might contribute to the increased FcRn binding of PFc29.

To elucidate how the Q311R mutation increased hFcRn binding when it was added to Fc-M428L, we generated the complex structure of hFcRn–PFc29 (M428L and Q311R), which revealed that the Q311R substitution created salt bridges with two hFcRn residues, E138 and E139 (distance of E138_hFcRn_–Q311R_PFc29_: 3.9 Å, E139_hFcRn_–Q311R_PFc29_: 4 Å) (Fig. 3c). Based on those structural analysis results, the interaction between PFc29 (Q311R/M428L) and hFcRn is likely strengthened by lateral displacement and additionally stabilized by the formation of two salt bridges.

### Trastuzumab-PFc29 had favorable physicochemical properties and exhibited enhanced antibody-dependent cellular cytotoxicity and complement-dependent cytotoxicity effector functions

Novel mutations introduced into an IgG antibody for the desired function might affect not only the structure but also the developability of the molecule. Therefore, careful evaluation of physicochemical properties is indispensable. We examined how the two mutations of the PFc29 variant with improved pH-dependent FcRn binding affected thermostability, glycan profiles, isoelectric point (pI), and potential immunogenicity. The melting temperature 1 (T_m_1) of trastuzumab-PFc29 was comparable to that of trastuzumab and 7 °C higher than that of trastuzumab-YTE (Table 2). Additionally, all the trastuzumab-Fc variants showed *N*-linked glycosylation profiles almost identical to those of trastuzumab (Supplementary Fig. 5a–d), and the pI value of trastuzumab-PFc29 was similar to that of clinical-grade trastuzumab (Herceptin®; Supplementary Fig. 6a–e).

**Table 2:** Thermostability analysis of trastuzumab and trastuzumab-Fc variants.

Sample	Tm1 (CH2)	Tm2 (Fab)	Tm3 (CH3)
Trastuzumab	71.3	80.0	82.0
Trastuzumab-YTE	64.4	79.4	81.4
Trastuzumab-LS	71.3	79.4	81.4
Trastuzumab-PFc29	70.1	79.4	81.4

**Fig. 4:**
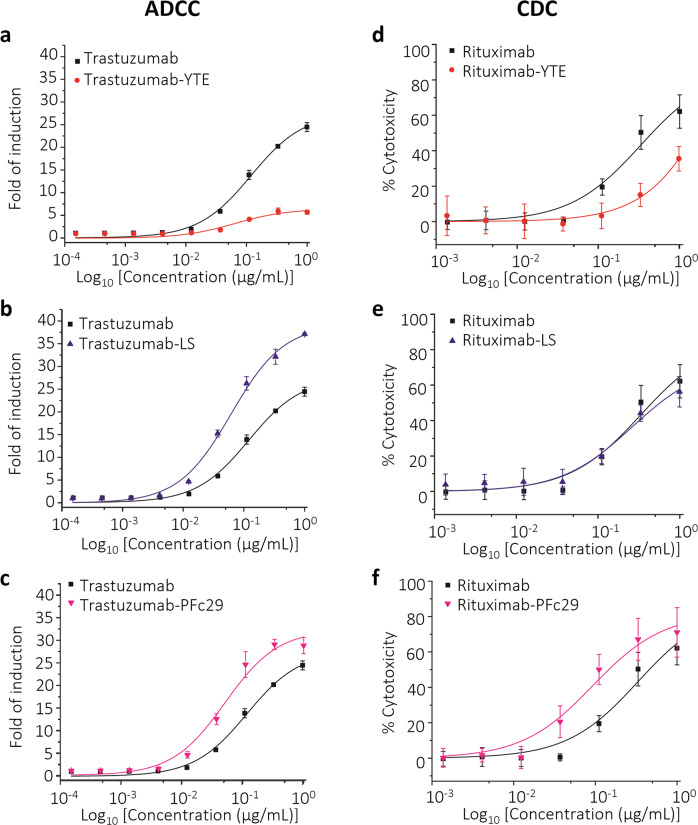
Effects of Fc variants on effector functions of IgG antibodies. The ADCC activities of trastuzumab-YTE **a**, trastuzumab-LS **b**, and trastuzumab-PFc29 **c** were compared with that of trastuzumab. Luminescence signals resulting from trastuzumab-Fc variants engaging with target cells (SKBR-3) and effector cells (FcRIIIa-158V-expressing engineered Jurkat) were measured. **d**–**f** The CDC activities of rituximab-YTE **d**, rituximab-LS **e**, and rituximab-PFc29 **f** were analyzed using normal human complements and Raji cells as target cells. Rituximab-containing wild-type Fc was used as the control.

After confirming the excellent physicochemical properties of trastuzumab-PFc29, we evaluated its effector function for clearing tumor cells. Antigen-bound IgG antibodies recruit immune leukocytes through hFcγR binding, and the interaction between IgG Fc and hFcγRs triggers subsequent effector functions such as ADCC and antibody-dependent cellular phagocytosis (ADCP) to clear defective cells. In addition, an association between serum complement C1q and the Fc region of human IgG initiates CDC to remove target cells^17,41^. To examine the effector function of antibodies containing the Fc variants, we analyzed their binding affinity for hFcγRs and hC1q along with in vitro cell-based assays (ADCC and CDC). In the ELISA analysis to evaluate hFcγR binding, trastuzumab-YTE showed significantly reduced binding affinity to all hFcγRs, in good agreement with earlier studies^19,42^. On the other hand, trastuzumab-PFc29 showed a highly improved binding affinity for hFcγRIIIa (V/F) compared to trastuzumab and a similar binding affinity for hFcγRI, hFcγRIIa (H/R), and hFcγRIIb (Supplementary Fig. 7a–f). To explore the antitumor ADCC activity of the trastuzumab-Fc variants, we used the SKBR-3 cell line and the engineered Jurkat effector cells provided in the ADCC Reporter Bioassay Core Kit (Promega). The results showed that trastuzumab-PFc29 and trastuzumab-LS exhibited higher ADCC activity than trastuzumab (Fig. 4a–c and Supplementary Table 4). The ADCC activity of trastuzumab-YTE was significantly lower than that of the other trastuzumab-Fc variants, which was consistent with previous studies (Fig. 4a–c and Supplementary Table 4)^42^.

Before evaluating the CDC activity, we analyzed the interaction between hC1q and the trastuzumab-Fc variants using ELISA. Surprisingly, trastuzumab-PFc29 exhibited significantly improved affinity for hC1q compared with the other trastuzumab-Fc variants (Supplementary Fig. 7g). Then, the CDC activity of trastuzumab-Fc variants was analyzed using a human serum complement mixture and SKBR-3 cells as target cells. Contrary to expectation, the CDC activity of all the trastuzumab and trastuzumab-Fc variants was low (data not shown). We reasoned that the deficient CDC activity of the trastuzumab-Fc variants in the Her2-overexpressing SKBR-3 cell line was due to inhibition of the CDC cascades by complement regulator proteins such as CD55 and CD59^43^. Therefore, we introduced the identified Fc mutations (YTE, LS, and PFc29) into rituximab, which uses CDC as its primary effector function for clearing CD20-overexpressing non-Hodgkin’s lymphoma B cells. After we produced rituximab and the rituximab-Fc variants in Expi293F cells, we analyzed their purity using SEC-HPLC and found no detectable aggregation peak (Supplementary Fig. 4m–q). Before the CDC activity analysis, we analyzed the hC1q binding of the rituximab-Fc variants using ELISA and found that all the rituximab-Fc variants showed hC1q binding similar to that of the trastuzumab-Fc variants (Supplementary Fig. 7g, h). The CDC activity of the rituximab-Fc variants was evaluated using a human serum complement mixture and the Raji cell line, and the results showed that rituximab-LS exhibited CDC activity similar to that of rituximab. On the other hand, rituximab-YTE, with its reduced hC1q binding, elicited much lower CDC activity than rituximab. In sharp contrast, rituximab-PFc29 exhibited the highest CDC activity (3.5-fold improved CDC activity compared to rituximab and rituximab-LS; Fig. 4d–f and Supplementary Table 4).

### In combination with effector function-silencing mutations, the two mutations of PFc29 removed FcγRIIIa and C1q binding while retaining pH-dependent hFcRn binding

In cases of a cytokine neutralizing antibody^44^, immune cell surface-antigen blocking antibody^45^, and immune effector cell-engaging bispecific antibody^46^, natural IgG Fc might induce side effects by activating immune leukocytes^41^. Therefore, it is highly desirable to remove effector functions such as ADCC, ADCP, and CDC in those cases. We evaluated the applicability of PFc29, which offers improved pH-dependent hFcRn binding, to therapeutic antibodies that require the removal of the hFcγRs and C1q bonds that mediate immune effector functions. First, to eliminate the hFcγRs and C1q binding of PFc29, we expressed and purified rituximab-Fc variants (rituximab-PFc29-LALA, rituximab-PFc29-LALAPG, rituximab-PFc29-TL, and rituximab-PFc29-LALATL) with Fc silencing mutations: L234A/L235A (LALA)^47^, L234A/L235A/P329G (LALAPG)^48^, T299L (TL), and L234A/L235A/T299L (LALATL)^47,49^. SEC-HPLC analysis confirmed that these rituximab-Fc variants were successfully produced and had excellent physicochemical properties without aggregation or multimer formation (Supplementary Fig. 4r–u).

**Fig. 5:**
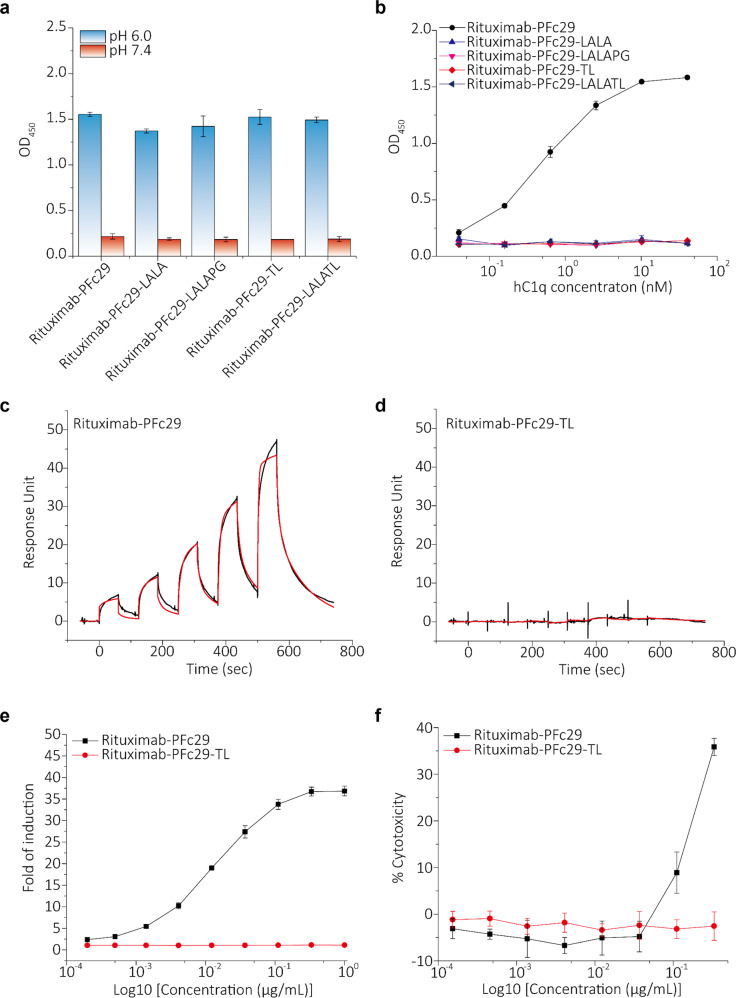
Analysis of the binding of Fc ligands (hFcRn, C1q, and hFcγRIIIa) and effector functions (ADCC and CDC) when the two mutations of PFc29 were combined with effector function-silencing mutations (LALA, LALAPG, or TL). **a** ELISA binding signals (OD_450_) upon binding rituximab-PFc29, rituximab-PFc29-LALA, rituximab-PFc29-LALAPG, rituximab-PFc29-TL, and rituximab-PFc29-LALATL to hFcRn at two pH conditions (pH 6.0 and pH 7.4) are represented as a bar graph. Errors bars indicate the standard deviations calculated from duplicate samples. **b** ELISA binding signals (OD_450_) upon binding rituximab-PFc29, rituximab-PFc29-LALA, rituximab-PFc29-LALAPG, rituximab-PFc29-TL, and rituximab-PFc29-LALATL to C1q are represented as a graph. **c**, **d** SPR sensorgrams showing the binding of rituximab-Fc variants to hFcγRIIIa. Interactions between hFcγRIIIa and rituximab-PFc29 **c** and rituximab-PFc29-TL **d** were analyzed using an SPR instrument. **e** ADCC activities of rituximab-PFc29-TL and rituximab-PFc29. Raji cells and engineered Jurkat cells expressing FcγRIIIa-158V were used as target cells and effector cells, respectively. **f** CDC activities of rituximab-PFc29-TL and rituximab-PFc29. Cytotoxicity was measured using Raji cells and normal human complements.

In the analysis of interactions between the resulting rituximab-Fc variants and Fc binding ligands such as hFcRn, hFcγR, and C1q, all the rituximab-Fc variants containing the putative Fc effector function-silencing mutations showed pH-dependent hFcRn binding very similar to that of rituximab-PFc29, but they also demonstrated an abolished binding affinity for hFcγRIIIa and C1q, a crucial receptor and a complement molecule for the ADCC and CDC effector functions, respectively (Fig. 5a–d and Supplementary Fig. 8a–c). Next, to examine whether the removal of hFcγRIIIa and hC1q binding silenced the effector functions of PFc29, we analyzed the ADCC and CDC activities of rituximab-PFc29-TL using human peripheral blood mononuclear cells (PBMCs) and a human serum complement mixture. The results indicate that the Fc effector functions of rituximab-PFc29-TL were successfully silenced (Fig. 5e, f), suggesting that PFc29 with effector function-silencing mutations can be applied to therapeutic antibodies in which abolished effector functions and improved serum persistence are desirable.

### Antibodies containing the PFc29 variant elicited T-cell responses similar to those elicited by wild-type antibodies

**Fig. 6:**
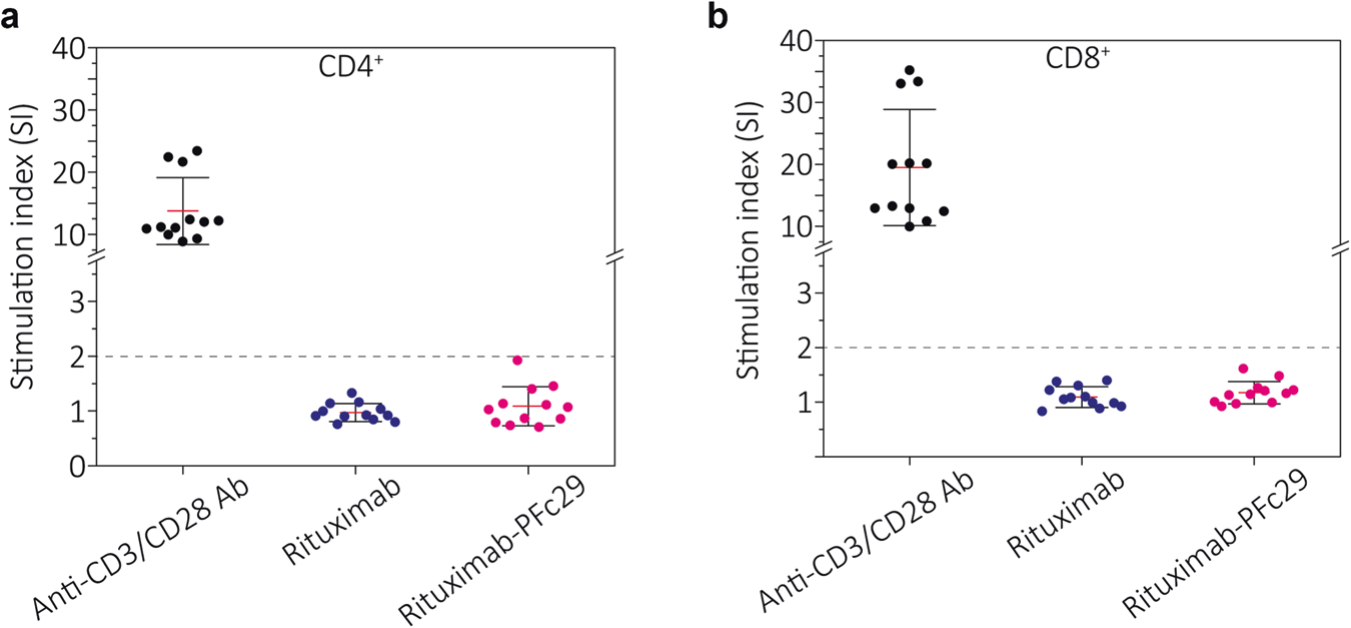
Analysis of T-cell activation induced by rituximab-PFc29. The immunogenicity of the rituximab-Fc variants was examined by measuring the proliferation of CD4+ or CD8+ T cells. **a**, **b** Scatter plots representing the proliferation stimulation index (SI) values of CD4+ (**a**) and CD8+ (**b**) T cells from four healthy donors upon incubation with rituximab, rituximab-PFc29, or anti-CD3/CD28 antibody. Red lines indicate the mean values, and black lines represent standard deviations. The black dashed line indicates statistical significance at SI ≥2.0 and response at SI ≥2.0, which was considered positive in this study.

To investigate whether the two mutations of PFc29 (Q311R and M428) induce a T-cell response, 15-amino acid peptides from wild-type Fc and the PFc29 variants were generated in silico. Their binding affinity to the 27 types of MHC II alleles, those most frequently found in the human population, were analyzed using the Immune Epitope Database^50^. In that analysis, we classified peptides into three groups (high, medium, and low) based on their predicted binding affinities to MHC class II on T cells, which are highly correlated with T-cell immunogenicity^51^. The in silico immunogenicity assessment results for 27 human leukocyte antigen (HLA) types revealed that peptides containing the M428L and Q311R mutations of PFc29 exhibited immunogenicity almost identical to or even lower than peptides derived from wild-type Fc (Supplementary Fig. 9a,b). Although some peptides containing Q311R had a slight binding affinity to MHC class II in the two HLA types, it was not expected to cause immunogenicity issues because the MHC class II binding affinities of those peptides were not high (Supplementary Fig. 9a, b). In good agreement with the in silico evaluation results, an in vitro immunogenicity analysis using T cells from four healthy donors showed that the stimulation index (SI: percentage of stimulated cells/mean percentage of unstimulated control cells) of rituximab-PFc29 was lower than 2 (considered a negative T-cell response^52^), which was similar to the SI value of rituximab-containing wild-type human Fc (Fig. 6 and Supplementary Fig. 10). These results clearly indicate that the use of IgG antibodies and Fc-fusion proteins with PFc29 (Q311R and M428L) had a very low likelihood of triggering immunogenicity issues in clinical practice.

## Discussion

Engineering the Fc–hFcRn interaction is a highly effective strategy to modulate the pharmacokinetic profiles of therapeutic IgG antibodies and to optimize their efficacy and the convenience of their administration. To prolong the serum circulating half-lives of IgG antibodies through hFcRn binding-mediated intracellular trafficking and recycling, both strong binding with hFcRn under intracellular endosomal pH conditions (pH 6.0) and efficient dissociation at serum pH (pH 7.4) are indispensable^36,53^. However, Fc engineering to improve hFcRn binding at pH 6.0 is accompanied by increased binding at pH 7.4 in most cases. Therefore, it is highly challenging to discover Fc variants with enhanced pH-dependent binding^36^. In this study, we screened Fc variants displayed on the bacterial inner membrane^32^ and successfully isolated the PFc29 variant with improved pH-dependent hFcRn binding (binding affinity similar to the YTE variant at pH 6.0 and more efficient dissociation than the YTE variant at pH 7.4). Our pharmacokinetic analysis in a cynomolgus monkey model showed that trastuzumab-PFc29 had a better pharmacokinetic profile than trastuzumab-Fc variants incorporating previously clinically validated serum half-life extension Fc variants (YTE or LS). In addition, trastuzumab-PFc29 had excellent physicochemical properties and elicited enhanced ADCC compared with trastuzumab containing wild-type Fc, and the two mutations of PFc29 augmented the CDC of rituximab.

To date, Fc variants with various binding affinities to hFcRn have been isolated to improve the serum half-lives of therapeutic IgG antibodies and therapeutic Fc-fusion proteins, and those works have improved our understanding of the relationship between the Fc–hFcRn interaction and in vivo serum persistence^25–27^. Borrok *et al*. reported that there might be a threshold affinity between an IgG antibody and hFcRn at pH 7.4 that would improve the circulating half-life of the antibody. It was suggested that an IgG antibody exhibiting an FcRn binding affinity exceeding the threshold affinity at pH 7.4 by a certain level might not show serum half-life improvement even if hFcRn binding was increased under endosomal pH conditions^53^. Lee et al. reported that a DHS variant (L309D/Q311H/N434S) with lower binding than the LS and YTE variants at both pH 6.0 and pH 7.4 exhibited a prolonged half-life in a humanized mouse model^27^. In contrast to DHS, PFc29, which exhibited hFcRn binding almost identical to that of YTE but lower binding at pH 7.4 (Fig. 1d), showed a better pharmacokinetic profile than LS and YTE in cynomolgus monkeys (Fig. 2c). Taken together, these results highlight the critical importance of efficiently dissociating IgG from hFcRn at pH 7.4 for the prolonged serum persistence of IgG antibodies.

When we annotated the identified mutations of PFc29 (Q311R and M428L) on the X-ray crystal structure for the rat IgG2a–rat FcRn complex, we found that the amino acids of rat IgG2a in positions equivalent to those mutations of PFc29 are Arg and Leu, which are the critical amino acids by which PFc29 improves hFcRn binding. Among the currently available X-ray crystallographic structures of the Fc–FcRn complex, the rat IgG2a–rat FcRn structure^9^ enabled us to elucidate the FcRn interaction in a pH-dependent manner and explain the stoichiometry when FcRn binds to IgG. In addition, it has been established that the interaction between rat IgG2a and rat FcRn is stronger than the human IgG–human FcRn interaction^37^. The structures of rat FcRn and human FcRn are very similar (Supplementary Fig. 11a), particularly the sequence corresponding to the binding epitope of human FcRn that interacts with the Q311R mutation of PFc29 (amino acid 133-FALNGEEFM-141), which is the same as that of native rat FcRn (Supplementary Fig. 11b). Therefore, it is likely that the amino acids that increase the binding affinity of rat IgG2a–rat FcRn (compared with that of human IgG–human FcRn) are the same as the substitution mutations identified in PFc29 (Supplementary Fig. 11c).

*N*-linked glycosylation appended to the IgG Fc region is critical for the binding of Fc to Fc ligands (FcγRs and C1q) and for determining the potency of effector functions (ADCC and CDC). However, the glycosylation profile of trastuzumab-PFc29 was almost identical to that of trastuzumab (Supplementary Fig. 5). In our previous Fc engineering works and single-molecule spectroscopic analysis study, we found that mutations in the CH2–CH3 interface region could affect the flexibility of the upper CH2 region^54^ and that the mutations changed the FcγRs binding affinity and therapeutic effector function^34^. Combining the results of our current study and previous works, we reason that the two mutations of PFc29, which are located very far from the putative binding epitope of FcγRIIIa or Clq binding^55,56^, might induce an overall conformational change of the CH2 domain that confers better binding to FcγRIIIa or Clq.

Introducing mutations to the Fc region to improve hFcRn binding can interfere with the effector functions of the antibody^42^. Our results, as well as those of the previous studies^19,42,57^, demonstrate that the three mutations of the YTE variant improve FcRn binding and reduce binding with hFcγRIIIa and hC1q, which significantly lowers effector functions such as ADCC and CDC (Fig. 4a, d). In sharp contrast, the antibody containing the PFc29 variant both improved pH-dependent FcRn binding and demonstrated excellent binding to hFcγRs and hC1q (Fig. 4c, f), illustrating that the PFc29 variant is a versatile Fc variant in which various beneficial functions of Fc are enhanced by introducing only two mutations. Unlike the YTE variant, which can be applied to only a small range of indications due to its low effector functions^21^, antibodies with the PFc29 variant could be used for indications that require effector functions, such as treatments for infectious and autoimmune diseases and cancer immunotherapy^8,17^. The significance of the presence and improvement of Fc effector functions in antibodies that target infectious diseases, including coronavirus disease (COVID-19), has recently been reported^58–60^. These reports strongly suggest that the Fc–hFcγR interaction is highly connected to the efficacy of antiviral antibody therapeutics, emphasizing the significance of developing antiviral antibodies with improved hFcγR binding. Along that line, we expect that PFc29, which exhibits extended circulating serum persistence as well as improved effector functions, could improve both the therapeutic efficacy and prophylactic effects of treatments for various viral diseases.

In conclusion, we have shown that the two mutations of PFc29 improved both the circulating serum half-life and the effector functions of an IgG antibody, and our biophysical characterization and pharmacokinetic profile evaluation of trastuzumab-PFc29 in primates provided an understanding of the correlation between the Fc–hFcRn interaction and the serum half-life of IgG antibodies. The engineered Fc variants will enable the development of next-generation antibodies and protein therapeutics with improved half-lives and efficacy, which will benefit many patients with cancer, infectious diseases, and autoimmune diseases.

## Supplementary information

Revised Supplementary information
