# Supplementary material for: An Fc variant with two mutations confers prolonged serum half-life and enhanced effector functions on IgG antibodies
Source: Exp Mol Med. 2022 Nov 1;54(11):1850–61. doi: 10.1038/s12276-022-00870-5 (PMC9628495; doi:10.1038/s12276-022-00870-5)
Supplement: Supplementary file 1 — Revised Supplementary information [file 12276_2022_870_MOESM1_ESM.pdf]

SUPPLEMENTARY INFORMATION

An Fc Variant with Two Mutations Confers Prolonged Serum Half-Life and Enhanced Effector Functions on IgG Antibodies

Sanghwan Ko[1,2], Sora Park[3], Myung Ho Sohn[3], Migyeong Jo[1,4], Byoung Joon Ko[3,5], Jung-Hyun Na[6], Hojin Yoo[3], Ae Lee Jeong[3], Kyungsoo Ha[3], Ju Rang Woo[3], Chungsu Lim[3], Jung Hyu Shin[3], Dohyun Lee[3], So-Young Choi[3,*], Sang Taek Jung[1,2,4,7,*]

[1]Department of Biomedical Sciences, Graduate School, Korea University, Seongbuk-gu, Seoul 02707, Republic of Korea

[2]Institute of Human Genetics, Korea University College of Medicine, Seoul, Republic of Korea

[3]New Drug Development Center, Osong Medical Innovation Foundation, 123, Cheongju, Chungcheongbuk-do, 28160, Republic of Korea

[4]BK21 Graduate Program, Department of Biomedical Sciences, Korea University College of Medicine, Seoul, Republic of Korea

[5]School of Biopharmaceutical and Medical Sciences, Sungshin Women's University, Seoul, 02844, Republic of Korea

[6]*Department of Pharmaceutical Engineering, Sangji University, Wonju, Gangwon-do, 26339, Republic of Korea*

[7]*Biomedical Research Center, Korea University Anam Hospital, Seoul, Republic of Korea*

***Correspondence to**: *Sang Taek Jung (sjung@korea.ac.kr) or So-Young Choi (soyoung@kbiohealth.kr)

Supplementary Fig. 1 Establishment of a bacterial display system for screening Fc variants with improved hFcRn binding. *E. coli* spheroplasts displaying Fc variants (wild-type Fc, Fc-LS, Fc-N434W, and Fc-M428L) reported in previous studies were labeled with 40 nM hFcRn-SA-Alexa 488 at pH 5.8 and pH 7.4 conditions. The bar graph represents the mean fluorescence intensity, and the error bars show the standard deviations calculated from triplicate samples.

Supplementary Fig. 2 Sequence alignment for wild-type Fc and isolated Fc variants (PFc29, PFc41, EFc29, and EFc41).

Supplementary Fig. 3 Solid ribbon structure showing the mutation sites of the isolated Fc variants. The identified mutations for EFc29 **a** and EFc41 **b** were overlaid on the structure of the Fc region of the human IgG crystal structure (PDB: 1HZH).

Supplementary Fig. 4 Size exclusion chromatography (SEC) analysis of trastuzumab and trastuzumab-Fc variants, aflibercept and aflibercept variants, and rituximab and rituximab variants. Each antibody variant (1 mg/ml) was injected into a BioSuit High-Resolution SEC column and developed in 1× PBS (pH 7.4) at a flow rate of 1 ml/min. Chromatograms for clinical grade trastuzumab (Herceptin®) **a**, trastuzumab prepared in-house **b**, trastuzumab-YTE **c**, trastuzumab-LS **d**, trastuzumab-PFc29 **e**, trastuzumab-PFc41 **f**, trastuzumab-EFc29 **g**, trastuzumab-EFc41 **h**, clinical grade aflibercept (Eylea®) **i**, aflibercept prepared in-house **j**, aflibercept-PFc29 **k**, aflibercept-PFc41 **l**, clinical grade

rituximab **m**, rituximab prepared in-house **n**, rituximab-YTE **o**, rituximab-LS **p**, rituximab-PFc29 **q**, rituximab-PFc29-LALA **r**, rituximab-PFc29-LALAPG **s**, rituximab-PFc29-TL **t**, and rituximab-PFc29-LALATL **u**.

Supplementary Fig. 5 Glycan profiles of trastuzumab-Fc variants. LC MS/MS spectra of trastuzumab **a**, trastuzumab-YTE **b**, trastuzumab-LS **c**, and trastuzumab-PFc29 **d**.

Supplementary Fig. 6 Capillary isoelectric focusing electrophoresis analysis to identify charge variants of trastuzumab-Fc produced electropherograms showing the isoelectric points of clinical grade trastuzumab (Herceptin®) **a**, trastuzumab prepared in-house **b**, trastuzumab-YTE **c**, trastuzumab-LS **d**, and trastuzumab-PFc29 **e**.

Supplementary Fig. 7 ELISA assays showing the binding of IgG-Fc variants to hFcγRs and hC1q. Binding of trastuzumab-Fc variants to hFcγRI **a**, hFcγRIIb **b**, hFcγRIIa-131H **c**, hFcγRIIa-131R **d**, hFcγRIIIa-158V **e**, hFcγRIIIa-158F **f**, and hC1q **g** were analyzed. **h** ELISA curve showing binding between rituximab-Fc variants and human C1q. Errors bars represent the standard deviations calculated from duplicate samples.

Supplementary Fig. 8 SPR sensorgrams showing the binding of rituximab-Fc variants to hFcγRIIIa. Interactions between hFcγRIIIa and rituximab-PFc29-LALA **a**, rituximab-PFc29-LALAPG **b**, and rituximab-PFc29-LALATL **c** were analyzed using an SPR instrument.

Supplementary Fig. 9 *In silico* immunogenicity analysis of Fc variants. The y-axis and x-axis indicate the input sequence and the 27 most frequently detected HLA types, respectively. The score for each peptide is provided as the percentile rank of the predicted binding affinity of the given peptide among a large number of peptides (15-mer window with an overlap of 14-mer). Red, orange, and yellow in the heatmap represent high (<2 in the percentile rank), medium (<5), and low (<10) affinities between a peptide and an MHC class II HLA type, respectively.

Supplementary Fig. 10 Analysis of T cell activation induced by rituximab-PFc29. The immunogenicity of rituximab-Fc variants was examined by measuring the proliferation of $CD4^+$ or $CD8^+$ T cells. **a** and **b** Density plots showing the proliferation of $CD4^+$ **a** and $CD8^+$ **b** T cells.

Supplementary Fig. 11 Comparison of Fc–FcRn structures and sequences between rat and human. **a** Structural alignment of the rat FcRn (orange)–rat Fc (green) complex structures (PDB: 1I1A) and human FcRn (olive) (PDB: 1EXU)–human Fc (silver) complex structures (PDB: 5JII). Structure-based sequence alignment of rat FcRn:human FcRn **b** and rat Fc:human Fc **c**. Blue-lined and red-lined boxes indicate the Fc binding site and FcRn binding site, respectively. Yellow or green sequences represent identical or similar amino acids in the sequence alignment, respectively. The numbers in **b** and **c** indicate the amino acid numbers based on the numbering scheme for human FcRn and human IgG Fc, respectively.

Supplementary Table 1. Plasmids used in this study.

Supplementary Table 2. Primers used in this study.

Supplementary Table 3. Equilibrium dissociation constants (K_D) and rate constants (k_{on} and k_{off}) for binding between trastuzumab-Fc variants and hFcRn at pH 6.0, and RU_{max} values of trastuzumab-Fc variants upon binding to hFcRn at pH 7.4, which were determined by an SPR analysis.

Supplementary Table 4. ADCC analysis of trastuzumab variants and CDC analysis of rituximab variants.

Materials and methods

Construction of plasmids

All primers used in this study are summarized in Supplementary Table 2. To construct plasmids that express human FcRn (hFcRn), genes encoding the extracellular region of the human FcRn α chain [1] and human β-2-microglobulin [1] were synthesized by Genscript (Scotch Plains, NJ, USA) and amplified using the appropriate pair of primers among SHK#34/SHK#35, SHK#36/SHK#37, SHK#38/SHK#39, and SHK#40/SHK#41 (Supplementary Table 2). The amplified PCR products were ligated into pMAZ-IgL-GlycoT [2] using the *Bss*HII / *Xba*I restriction enzyme sites. For pMAZ-hFcRn-FLAG-streptavidin-His, three PCR products, which were amplified using i) SHK#42/SHK#43 primers and the pcDNA3-FcRn-GST template, ii) SHK#44/SHK#45 primers and the pcDNA3-hFcRn-GST template, and iii) SHK#46/SHK#47 primers and the pMAZ-hFcγRIIIa-158V-streptavidin-His template [3], were assembled using two primers (SHK#42/SHK#47). Then, the assembled PCR products were ligated into pMAZ-IgL-GlycoT [2] using the *Bss*HII / *Xba*I restriction enzyme sites.

To construct rituximab heavy chains and light chains, the VH-CH1 and VL-CL genes of rituximab (Drugbank accession number: DB00073) were synthesized in Genscript (Scotch Plains, NJ, USA). The synthesized VH-CH1 was PCR amplified using primers (SHK#50/SHK#31) and assembled with Fc genes, which were amplified from pMAZ-IgH-GlycoT [2] by primer SHK#32/SHK#33 to construct full heavy chains. The synthesized light chain gene of rituximab was PCR amplified using two primers (SHK#48/SHK#49) for cloning into a pMAZ vector. The heavy chain and light chain genes were digested with

*Bss*HII / *Xba*I restriction enzymes and ligated into pMAZ-IgL-GlycoT [2] to generate pMAZ-IgH-rituximab and pMAZ-IgL-rituximab, respectively.

To construct plasmids for heavy chains of trastuzumab-Fc variants (YTE, LS, PFc29, PFc41, EFc29, and EFc41), rituximab-Fc variants (YTE, LS, and PFc29), or aflibercept-Fc variants (YTE, LS, PFc29, and PFc41), genes were generated by assembling two fragments (Fragment #1 and Fragment #2). Fragment #1 for the gene of the VH-CH1 region of IgG (trastuzumab or rituximab) or vascular endothelial growth factor receptor (VEGFR) was amplified using the proper primer set (SHK#30/SHK#31 for VH-CH1 of trastuzumab or rituximab and SHK#30/SHK#51 for VEGFR) and a template plasmid (pMAZ-IgH-GlycoT, pMAZ-IgH-rituximab, or gene-synthesized aflibercept [Drugbank accession number: DB08885]). For Fragment #2, each Fc variant gene (YTE, LS, PFc29, PFc41, EFc29, or EFc41) was amplified using a pair of primers (SHK#32/SHK#33) and assembled using two primers (SHK#29/SHK#33). This was followed by ligation of the assembled PCR products into pMAZ-IgH-GlycoT digested with *Bss*HII and *Xba*I restriction endonuclease sites to generate pMAZ-IgH-trastuzumab-YTE, pMAZ-IgH-trastuzumab-LS, pMAZ-IgH-trastuzumab-PFc29, pMAZ-IgH-trastuzumab-PFc41, pMAZ-IgH-trastuzumab-EFc29, pMAZ-IgH-trastuzumab-EFc41, pMAZ-IgH-rituximab-YTE, pMAZ-IgH-rituximab-LS, pMAZ-IgH-rituximab-PFc29, pMAZ-aflibercept-YTE, pMAZ-aflibercept-LS, pMAZ-aflibercept-PFc29, and pMAZ-aflibercept-PFc41. All the ligation products were transformed into *Escherichia coli* Jude1 (*F ′ [Tn10(Tetr) proAB+ lacIq Δ(lacZ)M15] mcrA Δ(mrr-hsdRMS-mcrBC) 80dlacZΔM15 ΔlacX74 deoR recA1 araD139 Δ (ara leu)7697 galU galK rpsL endA1 nupG*).

Preparation of human FcRn (hFcRn) and human FcγRs (hFcγRs)

All plasmids used in this study are summarized in Supplementary Table 1. Each pair of plasmids, pMAZ-hFcRn α chain-His/pMAZ-hβ2m, was co-transfected into Expi293F cells to express monomeric forms of hFcRn (monomeric human FcRn α chains complexed with human β2-microglobulin). Then, pcDNA3-hFcRn-GST [4], pMAZ-hFcγRIIa-131H-GST, pMAZ-hFcγRIIa-131R-GST, pMAZ-hFcγRIIIa-158V-GST, pMAZ-hFcγRIIIa-158F-GST, pMAZ-hFcγRIIb-GST, and pMAZ-hFcRn-FLAG-streptavidin-His were transfected separately into Expi293F cells to produce dimeric GST-fused forms of Fc receptors (hFcRn α chain-hβ2-microglobulin, hFcγRIIa-131H, hFcγRIIa-131R, hFcγRIIIa-158V, hFcγRIIIa-158F, and hFcγRIIb) and streptavidin-fused single-chain human FcRn (hFcRn-FLAG-streptavidin), respectively. The cells transfected with PEI-Max were cultured in GIBCO FreeStyle™ 293 expression medium for seven days. After harvesting by centrifugation at 6,000 × g for 10 min, the supernatants were mixed with 1/25 volume of 25× phosphate-buffered saline (PBS), filtered through a 0.2-μm bottle-top filter (MilliporeSigma Life Science, Burlington, MA, USA), and added to 1 ml of either Ni-NTA agarose resin or glutathione agarose 4B resin for binding to polyhistidine-tagged Fc receptors or GST-fused Fc receptors, respectively. Following overnight incubation at 4 °C and transfer of the resins into disposable polypropylene columns (Thermo Fisher Scientific), the Ni-NTA agarose resins were washed sequentially with 100 ml of 1× PBS, 25 ml of 10 mM imidazole buffer (pH 7.4), and 25 ml of 20 mM imidazole buffer (pH 7.4). Then, the monomeric and tetrameric FcRn proteins were eluted with 4 ml of 250 mM imidazole buffer (pH 7.4). GST-fused

dimeric hFcRn and hFcγRs bound to the glutathione agarose 4B resin were purified by washing with 10 ml of 1× PBS and elution with 4 ml of 50 mM Tris-HCl (pH 8.0) containing 10 mM GSH. Next, the buffer of the eluted hFcRn and hFcγRs was exchanged using Amicon Ultra-4 spin columns (3 kDa cutoff; MilliporeSigma Life Science, Burlington, MA, USA). The concentration and purity of the prepared Fc receptors were analyzed by measuring absorbance at 280 nm using a spectrophotometer (BioTek, Winooski, VT, USA) and by running the samples on 4–15% SDS-PAGE gels (Bio-Rad, Hercules, CA, USA), respectively.

Library construction

All primers used in this study are summarized in Supplementary Table 2. To construct an error-prone library (Library-E) consisting of Fc variants with random point mutations on the Fc region, the standard error-prone PCR technique [5] was used, and PCR products amplified using the SHK#3 and SHK#4 primers were ligated into *Sfi*I-digested pMopac12-NlpA-FLAG [3]. For the focused library (Library-F) of Fc variants with random saturation mutations in the FcRn binding site, three sub-libraries (L-1, L-2, and L-3) were constructed by assembling two fragments (Fragment A and Fragment B). Fragment A was PCR amplified using primers (SHK#1/SHK#13 for L-1, SHK#1/SHK#20 for L-2, and SHK#1/SHK#24 for L-3) and a template (pMopac12-NlpA-Fc-FLAG) [3]. For PCR amplification of Fragment B, the same template (pMopac12-NlpA-Fc-FLAG), sets of forward primers (SHK#14-19 for L-1, SHK#21-23 for L-2, SHK#25-28 for L-3), and a reverse primer (SHK#2) were used. After assembly PCR with Fragment A and Fragment B using the SHK#1/SHK#2

primers, the PCR products were ligated into pMopac12-NlpA-Fc-FLAG [3] using the *Sfi*I restriction enzyme sites, and the ligation products were transformed into *E. coli* Jude1.

E. coli culture conditions and spheroplasting

E. coli Jude1 inoculated into 25 ml of Terrific Broth (TB) supplemented with 2% (wt/vol) glucose and chloramphenicol (40 μg/ml) was incubated at 37 °C for 5 h with shaking at 250 rpm. After 100-fold dilution of culture broth in 100 ml of fresh TB medium supplemented with antibiotics as needed and further incubation at 37 °C until OD_{600} reached 0.6, the cultivated cells were incubated at 25 °C for 20 min and induced with 1 mM of isopropyl-1-thio-*D*-galactopyranoside for protein expression. Following cultivation of the cells at 25 °C for 5 h, an aliquot of the culture broth equivalent to 8 ml/OD_{600} was harvested by centrifugation at 12,000 × g. Then, the cells were washed twice in 1 ml of cold 10 mM Tris-HCl (pH 8.0) and resuspended in 1 ml of cold STE solution (0.5 M sucrose, 10 mM Tris-HCl, and 10 mM EDTA at pH 8.0). After incubation at 37 °C for 30 min on a rotator and centrifugation at 12,000 × g for 1 min, cells were resuspended in 1 ml of cold Solution A (0.5 M sucrose, 20 mM $MgCl_2$, and 10 mM 3-(N-morpholino)propanesulfonic acid (MOPS); pH 6.8) and incubated in 1 ml of Solution A supplemented with 1 mg/ml of hen egg lysozyme at 37 °C for 15 min. Following pelleting by centrifugation at 12,000 × g, the spheroplasts were resuspended in 1 ml of cold PBS.

High-throughput library screening using flow cytometry

First, 1 mg of tetrameric human single-chain FcRn-streptavidin-His (hFcRn-SA-His) was labeled with Alexa Fluor 488 fluorescent dye using an Alexa Fluor 488 Protein Labeling kit (Thermo Fisher Scientific) according to the manufacturer's guidelines. After dilution of 200 μl of the prepared spheroplast suspension in 800 μl of cold 1× PBS, the spheroplasts were added to 40 nM of tetrameric hFcRn-Alexa Fluor 488 and incubated in the dark at room temperature for 1 h with vigorous mixing on a rotator. The fluorescently labeled spheroplasts, which were pelleted by centrifugation at 12,000 × g for 1 min and washed in 1 ml of cold PBS, were resuspended in 30 ml of cold 1 × PBS, passed through a cell strainer (BD Biosciences, Franklin Lakes, NJ, USA) to remove aggregates, and sorted using an S3 Cell Sorter (Bio-Rad, Hercules, CA, USA) to recover the spheroplasts exhibiting the highest fluorescence intensity of 3%. The sorted spheroplasts were immediately re-sorted to improve sorting purity and then concentrated using an Amicon ultra-centrifugal filter 30K (MilliporeSigma Life Science, Burlington, MA, USA). The genes encoding the IgG Fc region were PCR rescued using two primers (SHK#1 and SHK#2), ligated into pMopac12-NlpA-Fc-FLAG using the *Sfi*I restriction enzyme sites, and transformed into *E. coli* Jude1. In the next rounds of flow cytometric sorting, 30 nM (second and third rounds) or 10 nM (fourth and fifth) tetrameric hFcRn-Alexa Fluor 488 (hFcRn-SA-Alexa 488) was used to label the spheroplasts, and highly fluorescent spheroplasts were sorted as in the first round of flow cytometric sorting.

Expression and purification of trastuzumab-, rituximab-, and aflibercept-Fc variants

All plasmids used in this study are summarized in Supplementary Table 1. A plasmid for an IgG heavy chain (pMAZ-IgH-GlycoT, pMAZ-IgH-trastuzumab-YTE, pMAZ-IgH-trastuzumab-LS, pMAZ-IgH-trastuzumab-PFc29, pMAZ-IgH-trastuzumab-PFc41 pMAZ-IgH-trastuzumab-EFc29, pMAZ-IgH-trastuzumab-EFc41, pMAZ-IgH-rituximab, pMAZ-IgH-rituximab-YTE, pMAZ-IgH-rituximab-LS, pMAZ-IgH-rituximab-PFc29, or pMAZ-IgH-rituximab-PFc41) was co-transfected with a plasmid for an IgG light chain (pMAZ-IgL-GlycoT or pMAZ-IgL-rituximab) into Expi293F cells using PEI-MAX. For the expression of aflibercept and the aflibercept-Fc variants, pMAZ-aflibercept, pMAZ-aflibercept-PFc29, or pMAZ-aflibercept-PFc41 was transfected into Expi293F cells using the same transfection reagent and conditions. After incubation in GIBCO FreeStyle™ 293 expression medium at 37 °C with 8% CO_2 for 7 days and centrifugation at 2,000 x g for 10 min, the culture supernatants were mixed with 40 ml of 25× PBS per liter of culture, filtered through a 0.2-µm bottle-top filter, and added to 1 ml of Protein A agarose. Following incubation overnight at 4 °C, transfer of the resin into a polypropylene column, and washing of the column twice with 10 ml of 1× PBS, the purified trastuzumab-, rituximab-, and aflibercept-Fc variants were eluted using 3 ml of 100 mM glycine-HCl (pH 2.7), which was immediately neutralized by collecting the eluate in a tube containing 1 ml of 1 M Tris (pH 8.0). After exchanging the buffer with 1× PBS and concentration using Amicon Ultra-4 spin columns with a 3-kDa cutoff filter, the purities of the prepared proteins were evaluated by analysis on 4–15% resolving SDS-PAGE gels.

Size exclusion chromatography analysis

For the size exclusion analysis, 1 mg/ml of trastuzumab-, rituximab-, or aflibercept-Fc variants in PBS was loaded into a BioSuit High-Resolution SEC column (7.5 mm × 300 mm, 10 µm) in a Waters Alliance 2695 high performance liquid chromatography (Waters, Milford, MA, USA). Each sample was developed in 1× PBS at a flow rate of 1 ml/min using the isocratic elution condition, and the elution signal was detected using a UV/Vis detector at 280 nm.

Molecular modeling of Fc variant–hFcRn complexes

Before we modeled the Fc variant–hFcRn complexes, we used SWISS-MODEL [6] to generate homology models for PFc29 and another Fc variant (M428L) using the crystal structure of wild-type Fc (PDB code: 4Q7D) [7] as a template. To determine the protonation state of the Fc variants at pH 6.0, the homology models for wild-type Fc (PDB code 4Q7D) were processed by *H++* [8] to add or eliminate hydrogen atoms on the amino acids. Discovery Studio (Dassault Systèmes, Vélizy-Villacoublay, France) was used to model the complex structures of the *H++* processed Fc variants, human FcRn-α chain, and β2-microglobulin. Ternary complex structures of the YTE-Fc, FcRn, β2-microglobulin, and serum albumin (PDB code: 4NOU) were used as a template for the complex modeling.

Thermostability analysis

Thermostability was analyzed using a Nano differential scanning calorimeter (DSC, TA Instruments, New Castle, DE, USA) with a 1 °C/min scan rate. First, 650 µl of protein (1 mg/ml) in 1× PBS was loaded into the sample cell of the Nano DSC, and the reference cell

was filled with 1× PBS buffer. After 10 min of equilibration at 10 °C and thermoscanning in the range from 10 to 110 °C, the DSC profiles were monitored using NanoAnalyze Data Analysis Version 3.7.5 software. After subtracting buffer background and normalizing the thermogram to the molar concentration of the protein, the data were fitted using a non-two-state model with three peaks to represent the values of T_m.

***N*-glycan profiling**

All experimental procedures for glycan release, labeling, and enrichment were based on a previous study [9]. Briefly, 25 µl of sample was mixed with 6 µl of 5% Rapi-gest™ SF (Waters) in water, heated for 3 min at 90 °C, cooled to room temperature, and mixed with 1.2 µl of GlycoWorks Rapid PNGase F (Promega, Madison, WI, USA). After incubation for 5 min at 50 °C and cooling to 25 °C for 3 min, the sample was combined with 12 µl of labeling solution for 5 min of incubation at 25 °C. Then, the samples were diluted in 385 µl of acetonitrile solution, and hydrophilic interaction liquid chromatography (HILIC) solid phase extraction (SPE) enrichment was conducted using a Waters GlycoWorks HILIC µElution plate. The plates were washed with 200 µl of water and conditioned with 200 µl of 85% acetonitrile. After conditioning, sample loading, and washing with 600 µl of washing solution (1:9:90 [v/v], formic acid:water:acetonitrile), the samples were eluted with three volumes of 30 µl elution buffer (200 mM ammonium acetate in 5% acetonitrile). To separate the glycans, an eluent A (50 mM ammonium formate, pH 4.5) and eluent B (100% acetonitrile) were prepared. Separation was accomplished by linear gradient elution for 35 min from 25% to 46% of eluent B at a flow rate of 0.4 ml/min using a Waters

ACQUITY I class UPLC system equipped with an ACQUITY UPLC Glycan BEH amide column (2.1 mm × 150 mm, 1.7 µm; Waters, Milford, MA, USA). The individual glycans were detected by excitation and emission at 264 nm and 425 nm, respectively.

Capillary isoelectric focusing electrophoresis

The samples were focused with a PA800 plus CE (Beckman Coulter, Brea, CA, USA) using an ampholyte Isoelectric point (pI) gradient from 3 to 10. All pretreatment was performed with a Capillary Isoelectric Focusing kit (Beckman Coulter, Bera, CA, USA). The sample was injected for 99 sec at 25 psi and focused for 15 min at 25 kV under normal polarity. Then, separation was performed for 30 min at 30 kV under normal polarity. The other capillary isoelectric focusing (cIEF) parameter settings and experimental procedures were based on the manufacturer's manual.

ELISA analysis

For the ELISA analysis, 50 µl of 4 µg/ml of HER2 were diluted in 0.05 M Na_2CO_3 (pH 9.6) and coated onto a flat-bottom, polystyrene, high-bind, 96-well microplate (Corning, Corning, NY, USA) by incubation at 4 °C for 16 hours. We added 4% skim milk in 1× PBS at pH 7.4 to the plate for blocking and incubated it at room temperature for one hour. After washing the plate four times with 180 µl of 0.05% PBST (1× PBS and 0.05% Tween20) at pH 7.4, 50 µl of IgG variants were added and incubated at room temperature for one hour. Subsequently, the plate was washed four more times, and 50 µl of dimeric hFcγRs (I, IIb, IIa(H/R), IIIa(V/F)) or hC1q (MilliporeSigma Life Science) serially diluted in 1% skim milk in

1× PBS at pH 7.4 was added. Then, 50 µl of HRP-conjugated antibodies; mouse anti-His-HRP conjugate (1:10,000) for detecting hFcγRI-His; goat anti-GST-HRP conjugate (1:5,000) for detecting hFcγRIIa-131H-GST, hFcγRIIa-131R-GST, hFcγRIIIa-158V-GST, hFcγRIIIa-158F-GST, and hFcγRIIb-GST; or sheep anti-hC1q-HRP (1:400) for detecting hC1q were added. After incubation at room temperature for one hour, four cycles of washing, and the addition of 50 µl of 1-Step Ultra TMB-ELISA Substrate Solution (Thermo Fisher Scientific), 50 µl of 2 M H_2SO_4 was added, and the absorbance at 450 nm was analyzed using an Epoch Microplate Spectrophotometer (BioTek).

***In silico* immunogenicity prediction**

The immunogenicity of the engineered Fc variants was predicted using the Immune Epitope Database (IEDB, http://tools.iedb.org/mhcii, ver 2.22). To analyze the binding between 15-mer peptides resulting from the amino acid sequence of wild-type IgG1 Fc (trastuzumab and rituximab Fc) or the PFc29 variant and broad spectrum alleles of MHC class II in the human population, the 27 most frequently detected HLA types (DPA1*01:03/DPB1*02:01, DPA1*01:03/DPB1*04:01, DPA1*02:01/DPB1*01:01, DPA1*02:01/DPB1*05:01, DPA1*02:01/DPB1*14:01, DPA1*03:01/DPB1*04:02, DQA1*01:01/DQB1*05:01, DQA1*01:02/DQB1*06:02, DQA1*03:01/DQB1*03:02, DQA1*04:01/DQB1*04:02, DQA1*05:01/DQB1*02:01, DQA1*05:01/DQB1*03:01, DRB1*01:01, DRB1*03:01, DRB1*04:01, DRB1*04:05, DRB1*07:01, DRB1*08:02, DRB1*09:01, DRB1*11:01, DRB1*12:01, DRB1*13:02, DRB1*15:01, DRB3*01:01, DRB3*02:02, DRB4*01:01, and DRB5*01:01) were set as the reference option for IEDB

analysis. The output results for the percentile rank (PR) of the resulting peptides displaying binding affinity for a wide range of MHC II alleles were classified into three groups, high (PR<2), medium (2<PR<5), and low (5<PR<10), and converted to our own visualization interface to show a colorful heatmap (red, orange, and yellow indicate high, medium, and low PRs, respectively).

In vitro **immunogenicity assessment**

To determine the immunogenicity of the rituximab-Fc variants, peripheral blood mononuclear cells isolated from four healthy donors were treated with DNase and cultured overnight at 37 °C in a 5% CO_2 incubator. The next day, the cells were harvested and incubated with FcR Blocking Reagent (Miltenyi Biotec, Bergisch Gladbach, Germany) at 4 °C for 5 min, and $CD14^+$ cells were isolated using CD14 MicroBeads (Miltenyi Biotec, Bergisch Gladbach, Germany). Then, $CD4^+$ and $CD8^+$ T cells were isolated using CD4 and CD8 MicroBeads (Miltenyi Biotec, Bergisch Gladbach, Germany). Dendritic cells (DCs) were differentiated from $CD14^+$ cells in a culture of AIM-V medium containing GM-CSF and IL-4 for seven days. After adding rituximab-Fc variants or anti-CD3/anti-CD28 antibodies during the culture of DCs for 24 h, the maturation of DCs was induced by adding IL-6, IL-1b, TNF-α, and PGE2 to AIM-V medium for an additional 24 h of incubation at 37 °C [10]. The immature and mature DCs were co-cultured with CFSE-labeled $CD4^+$ or $CD8^+$ T cells for six days, and the proliferation of $CD4^+$ or $CD8^+$ T cells was examined using FACSCantoII (BD Biosciences, Franklin Lakes, NJ, USA). Immunogenicity was expressed as a stimulation index as follows: simulation index = sample well/baseline.

Supplementary Fig. 1 Establishment of a bacterial display system for screening Fc variants with improved hFcRn binding. *E. coli* spheroplasts displaying Fc variants (wild-type Fc, Fc-LS, Fc-N434W, and Fc-M428L) reported in previous studies were labeled with 40 nM hFcRn-SA-Alexa 488 at pH 5.8 and pH 7.4 conditions. The bar graph represents the mean fluorescence intensity, and the error bars show the standard deviations calculated from triplicate samples.

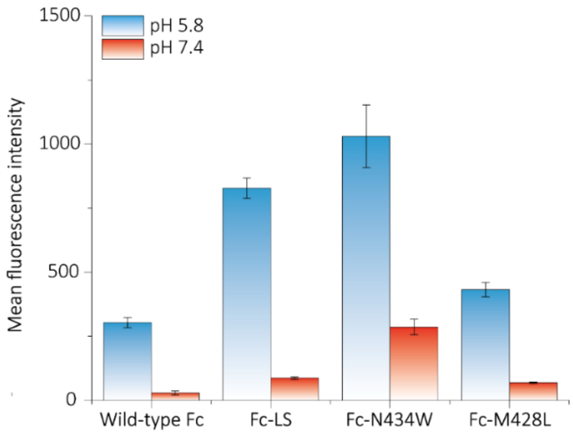

Supplementary Fig. 2 Sequence alignment for wild-type Fc and isolated Fc variants (PFc29, PFc41, EFc29, and EFc41).

```
                    230       240       250       260       270       280       290
Wild type Fc DKTHTCPPCPAPELLGGPSVFLFPPKPKDTLMISRTPEVTCVVVDVSHEDPEVKFNWYVDGVEVHNAKTKPREEQY
PFc29        ----------------------------------------------------------------------------
PFc41        ----------------------------------------------------------------------------
EFc29        -------L---------------------------------------------------------------L----
EFc41        -------L-----F----------------------------------D---------------------------

              300       310       320       330       340       350       360       370
Wild type Fc NSTYRVVSVLTVLHQDWLNGKEYKCKVSNKALPAPIEKTISKAKGQPREPQVYTLPPSRDELTKNQVSLTCLVKGF
PFc29        --------------R-------------------------------------------------------------
PFc41        ------------G---------------------------------------------------------------
EFc29        --------------------------------------------------------------A----G--------
EFc41        ---------------------------------------------L------------------------------

                  380       390       400       410       420       430       440
Wild type Fc YPSDIAVEWESNGQPENNYKTTPPVLDSDGSFFLYSKLTVDKSRWQQGNVFSCSVMHEALHNHYTQKSLSLSPGK
PFc29        -------------------------------------------------------L-------------------
PFc41        -------------------------------------------------------L-------------------
EFc29        -------------------------------------------------------L-------------------
EFc41        ---------------D-----A---------------------------------L-------------------
```

Supplementary Fig. 3 Solid ribbon structure showing the mutation sites of the isolated Fc variants. The identified mutations for EFc29 **a** and EFc41 **b** were overlaid on the structure of the Fc region of the human IgG crystal structure (PDB: 1HZH).

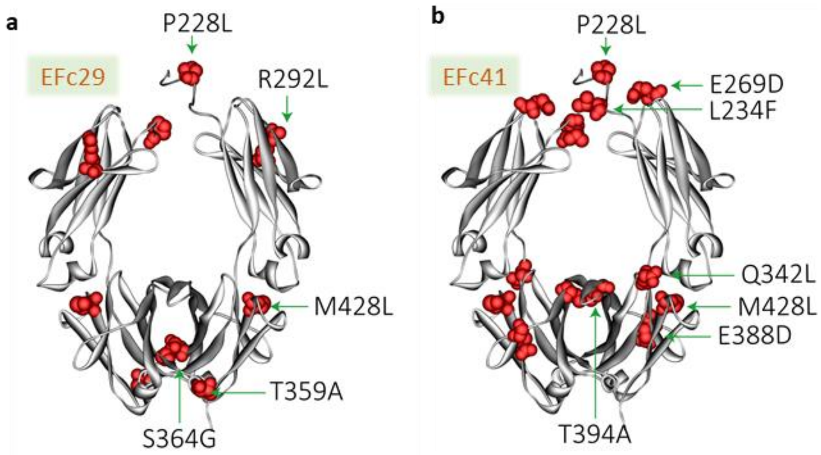

Supplementary Fig. 4 Size exclusion chromatography (SEC) analysis of trastuzumab and trastuzumab-Fc variants, aflibercept and aflibercept variants, and rituximab and rituximab variants. Each antibody variant (1 mg/ml) was injected into a BioSuit High-Resolution SEC column and developed in 1× PBS (pH 7.4) at a flow rate of 1 ml/min. Chromatograms for clinical grade trastuzumab (Herceptin®) **a**, trastuzumab prepared in-house **b**, trastuzumab-YTE **c**, trastuzumab-LS **d**, trastuzumab-PFc29 **e**, trastuzumab-PFc41 **f**, trastuzumab-EFc29 **g**, trastuzumab-EFc41 **h**, clinical grade aflibercept (Eylea®) **i**, aflibercept prepared in-house **j**, aflibercept-PFc29 **k**, aflibercept-PFc41 **l**, clinical grade rituximab **m**, rituximab prepared in-house **n**, rituximab-YTE **o**, rituximab-LS **p**, rituximab-PFc29 **q**, rituximab-PFc29-LALA **r**, rituximab-PFc29-LALAPG **s**, rituximab-PFc29-TL **t**, and rituximab-PFc29-LALATL **u**.

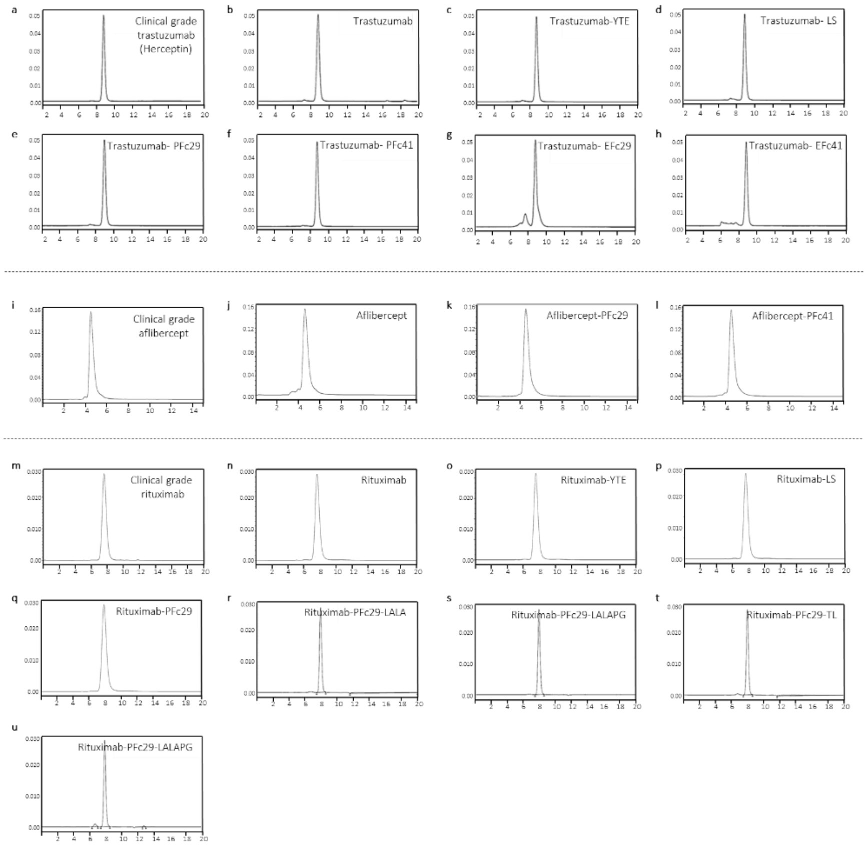

Supplementary Fig. 5 Glycan profiles of trastuzumab-Fc variants. LC MS/MS spectra of trastuzumab **a**, trastuzumab-YTE **b**, trastuzumab-LS **c**, and trastuzumab-PFc29 **d**.

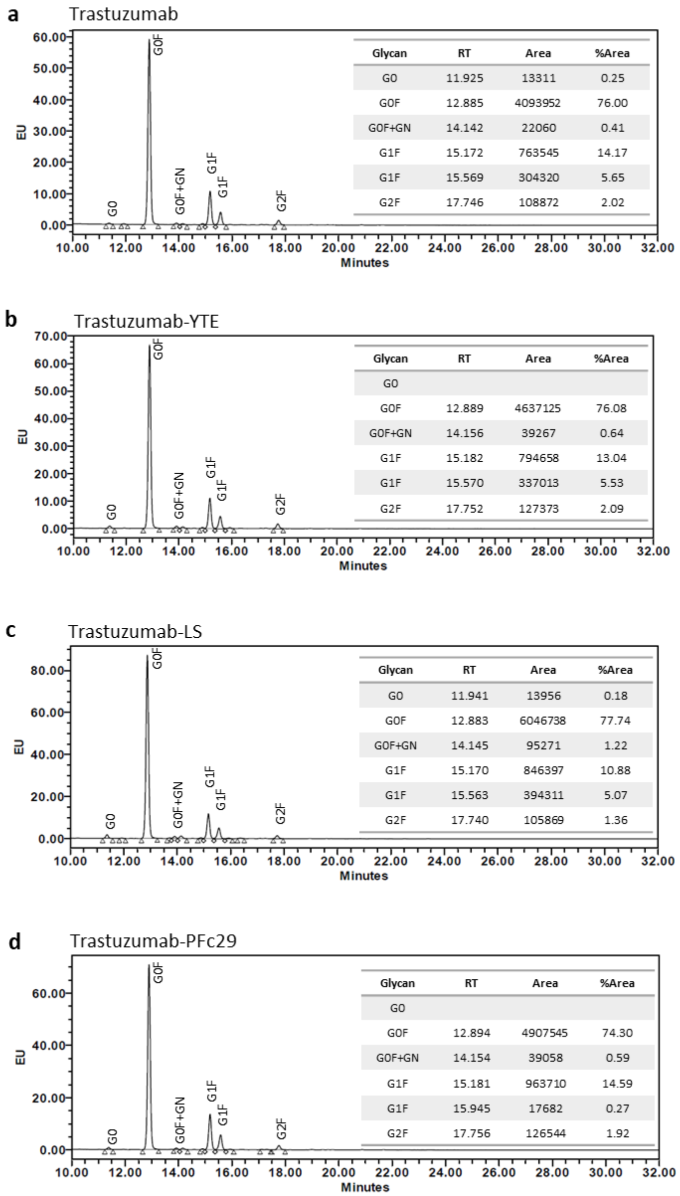

a Trastuzumab

Glycan	RT	Area	%Area
G0	11.925	13311	0.25
G0F	12.885	4093952	76.00
G0F+GN	14.142	22060	0.41
G1F	15.172	763545	14.17
G1F	15.569	304320	5.65
G2F	17.746	108872	2.02

b Trastuzumab-YTE

Glycan	RT	Area	%Area
G0			
G0F	12.889	4637125	76.08
G0F+GN	14.156	39267	0.64
G1F	15.182	794658	13.04
G1F	15.570	337013	5.53
G2F	17.752	127373	2.09

c Trastuzumab-LS

Glycan	RT	Area	%Area
G0	11.941	13956	0.18
G0F	12.883	6046738	77.74
G0F+GN	14.145	95271	1.22
G1F	15.170	846397	10.88
G1F	15.563	394311	5.07
G2F	17.740	105869	1.36

d Trastuzumab-PFc29

Glycan	RT	Area	%Area
G0			
G0F	12.894	4907545	74.30
G0F+GN	14.154	39058	0.59
G1F	15.181	963710	14.59
G1F	15.945	17682	0.27
G2F	17.756	126544	1.92

Supplementary Fig. 6 Capillary isoelectric focusing electrophoresis analysis to identify charge variants of trastuzumab-Fc produced electropherograms showing the isoelectric points of clinical grade trastuzumab (Herceptin®) **a**, trastuzumab prepared in-house **b**, trastuzumab-YTE **c**, trastuzumab-LS **d**, and trastuzumab-PFc29 **e**.

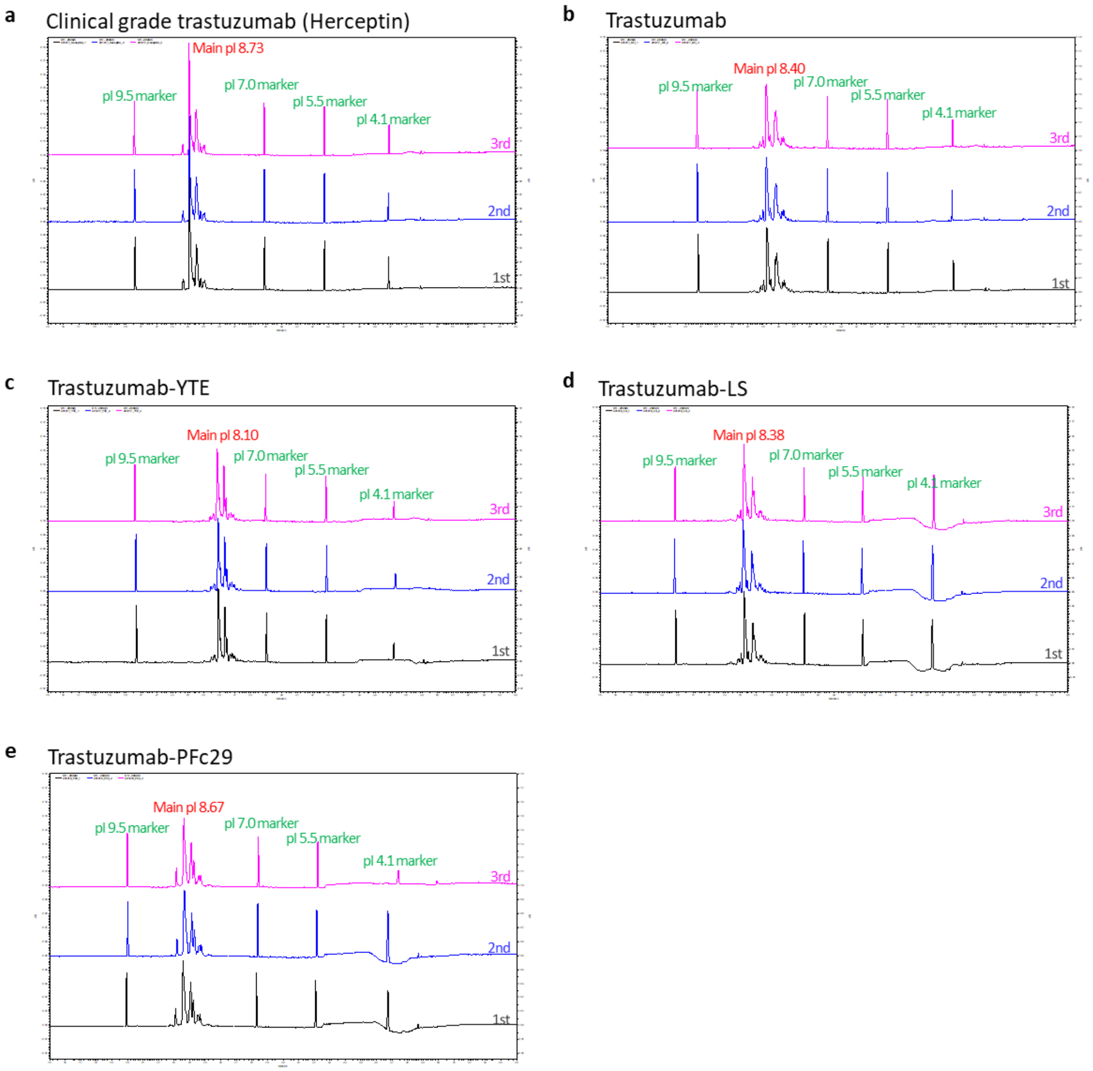

Supplementary Fig. 7 ELISA assays showing the binding of IgG-Fc variants to hFcγRs and hC1q. Binding of trastuzumab-Fc variants to hFcγRI **a**, hFcγRIIb **b**, hFcγRIIa-131H **c**, hFcγRIIa-131R **d**, hFcγRIIIa-158V **e**, hFcγRIIIa-158F **f**, and hC1q **g** were analyzed. **h** ELISA curve showing binding between rituximab-Fc variants and human C1q. Errors bars represent the standard deviations calculated from duplicate samples.

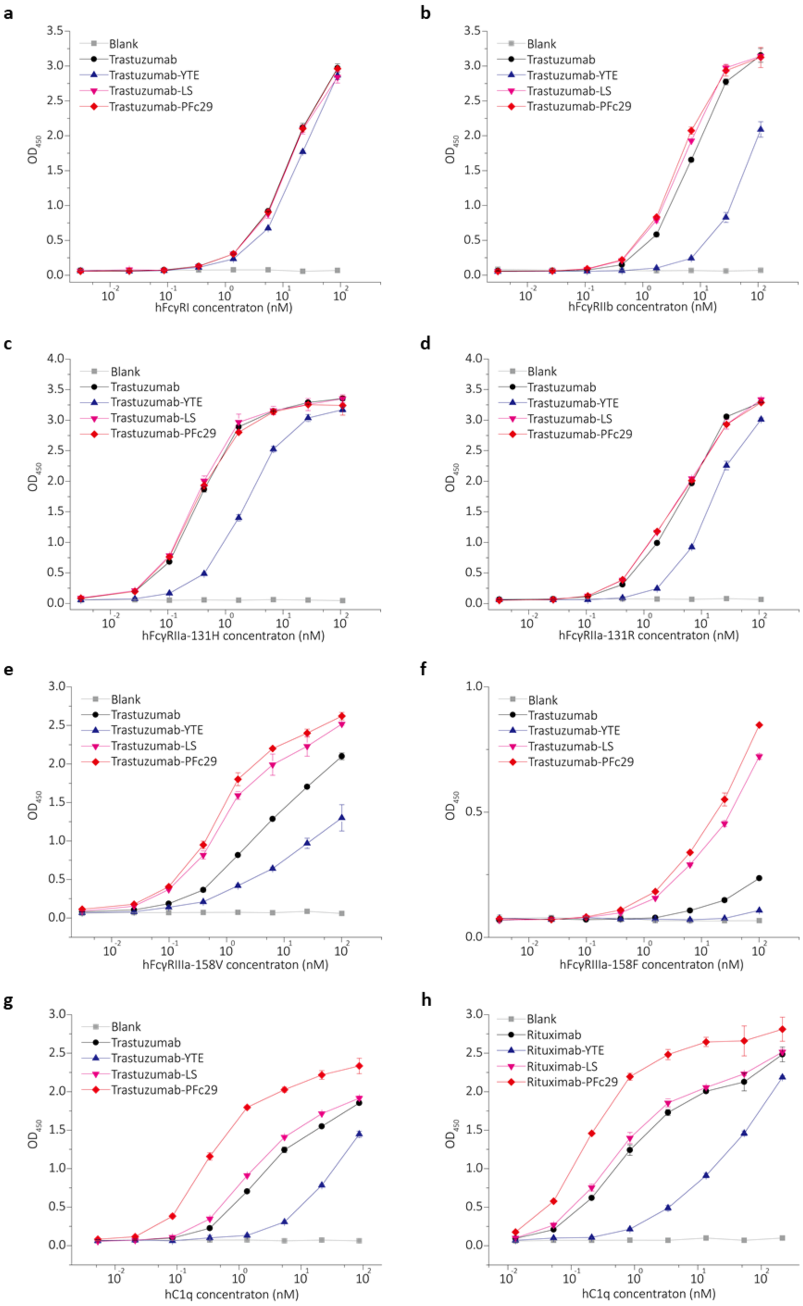

Supplementary Fig. 8 SPR sensorgrams showing the binding of rituximab-Fc variants to hFcγRIIIa. Interactions between hFcγRIIIa and rituximab-PFc29-LALA **a**, rituximab-PFc29-LALAPG **b**, and rituximab-PFc29-LALATL **c** were analyzed using an SPR instrument.

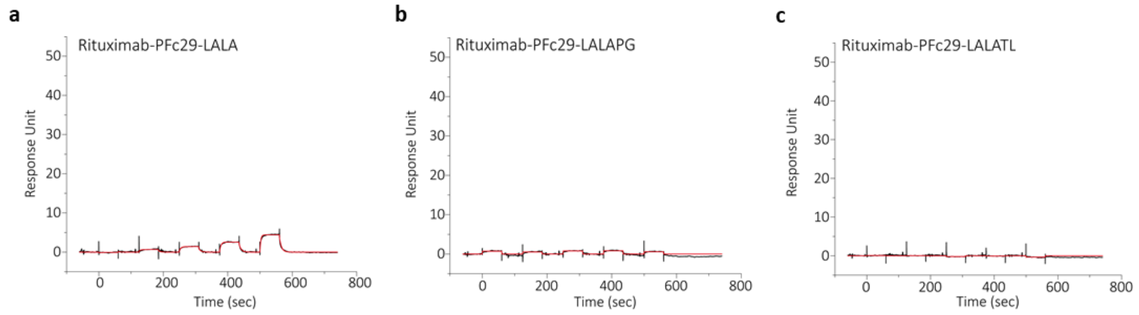

Supplementary Fig. 9 *In silico* immunogenicity analysis of Fc variants. The y-axis and x-axis indicate the input sequence and the 27 most frequently detected HLA types, respectively. The score for each peptide is provided as the percentile rank of the predicted binding affinity of the given peptide among a large number of peptides (15-mer window with an overlap of 14 mer). Red, orange, and yellow in the heatmap represent high (<2 in the percentile rank), medium (<5), and low (<10) affinities between a peptide and an MHC class II HLA type, respectively.

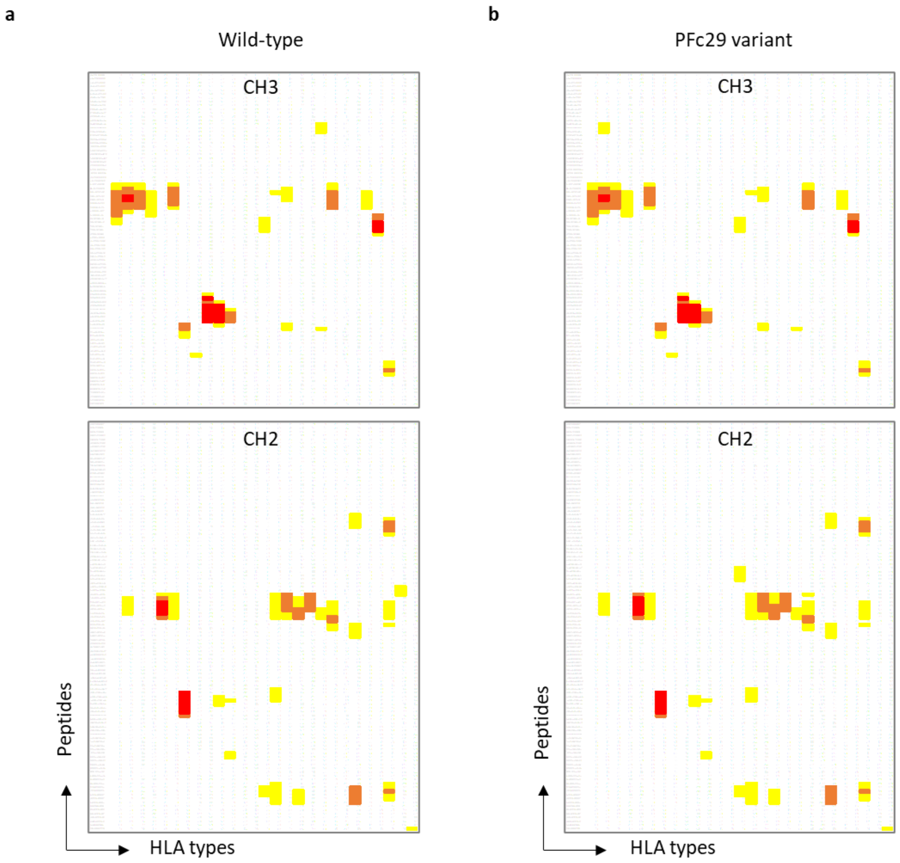

Supplementary Fig. 10 Analysis of T cell activation induced by rituximab-PFc29. The immunogenicity of rituximab-Fc variants was examined by measuring the proliferation of CD4$^+$ or CD8$^+$ T cells. **a** and **b** Density plots showing the proliferation of CD4$^+$ **a** and CD8$^+$ **b** T cells.

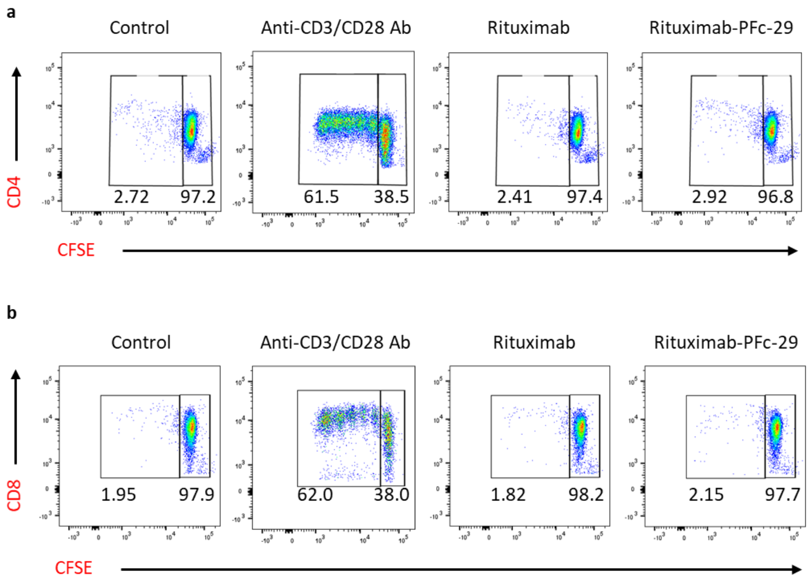

Supplementary Fig. 11 Comparison of Fc–FcRn structures and sequences between rat and human. **a** Structural alignment of the rat FcRn (orange)–rat Fc (green) complex structures (PDB: 1I1A) and human FcRn (olive) (PDB: 1EXU)–human Fc (silver) complex structures (PDB: 5JII). Structure-based sequence alignment of rat FcRn:human FcRn **b** and rat Fc:human Fc **c**. Blue-lined and red-lined boxes indicate the Fc binding site and FcRn binding site, respectively. Yellow or green sequences represent identical or similar amino acids in the sequence alignment, respectively. The numbers in **b** and **c** indicate the amino acid numbers based on the numbering scheme for human FcRn and human IgG Fc, respectively.

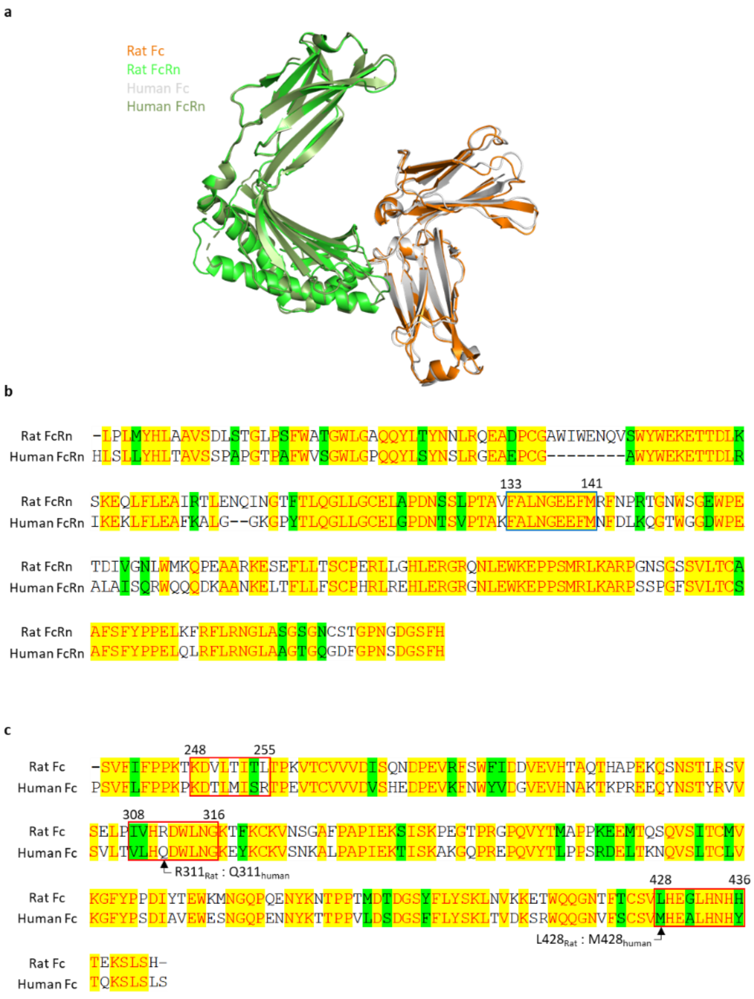

Supplementary Table 1. Plasmids used in this study.

Plasmid name	Relevant characteristics	Reference or source
pMopac12-NlpA-Fc-FLAG	Cm^r, *lac* promoter, *tetA* gene, *Fc* gene, C-terminal FLAG tag	[3]
pMopac12-NlpA-Fc-YTE-FLAG	Cm^r, *lac* promoter, tetA gene, *YTE mutant Fc* gene, C-terminal FLAG tag	Current study
pMopac12-NlpA-Fc-LS-FLAG	Cm^r, *lac* promoter, tetA gene, *LS mutant Fc* gene, C-terminal FLAG tag	Current study
pMopac12-NlpA-Fc-N434W-FLAG	Cm^r, *lac* promoter, tetA gene, *M434W mutant Fc* gene, C-terminal FLAG tag	Current study
pMopac12-NlpA-Fc-M428L-FLAG	Cm^r, lac promoter, tetA gene, *M428L mutant Fc* gene, C-terminal FLAG tag	Current study
pMAZ-IgH-GlycoT	*Trastuzumab H chain* gene in pMAZ-IgH-H23	[2]
pMAZ-IgL-GlycoT	*Trastuzumab L chain* gene in pMAZ-IgH-H23	[2]
pMAZ-hFcRn α chain-His	*Human FcRn α chain-His* gene in pMAZ-IgL	Current study
pMAZ-hβ2m	*Human β2- microglobulin* gene in pMAZ-IgL	Current study
pcDNA3-hFcRn-GST	Amp^r *CMV* promoter, human *FcRn α chain-GST and human beta 2 macroglobulin* gene in pcDNA3	[4]
pMAZ-hFcRn-FLAG-Streptavidin-His	*Human FcRn-FLAG-Streptavidin-His* gene in pMAZ-IgL	Current study
pMAZ-hFcγRIIa-131H-GST	*Human FcγRIIa$_{131H}$-GST* gene in pMAZ-IgL	[11]
pMAZ-hFcγRIIa-131R-GST	*Human FcγRIIa$_{131R}$-GST* gene in pMAZ-IgL	[11]
pMAZ-hFcγRIIIa-158V -GST	*Human FcγRIIIa$_{158V}$-GST* gene in pMAZ-IgL	[11]
pMAZh-FcγRIIIa-158F-GST	*Human FcγRIIIa$_{158F}$-GST* gene in pMAZ-IgL	[11]
pMAZ-hFcγRIIb -GST	*Human FcγRIIb-GST* gene in pMAZ-IgL	[11]
pMAZ-IgH-trastuzumab-YTE	*Trastuzumab YTE mutant H chain* gene in pMAZ-IgL	Current study
pMAZ-IgH-trastuzumab-LS	*Trastuzumab LS mutant H chain* gene in pMAZ-IgL	Current study
pMAZ-IgH-trastuzumab-PFc29	*Trastuzumab PFc29 mutant H chain* gene in pMAZ-IgL	Current study

pMAZ-IgH-trastuzumab-PFc41	*Trastuzumab PFc41 mutant H chain* gene in pMAZ-IgL	Current study
pMAZ-IgH-trastuzumab-EFc29	*Trastuzumab EFc29 mutant H chain* gene in pMAZ-IgL	Current study
pMAZ-IgH-trastuzumab-EFc41	*Trastuzumab EFc41 mutant H chain* gene in pMAZ-IgL	Current study
pMAZ-IgL-rituximab	*Rituximab L chain* gene in pMAZ-IgL	Current study
pMAZ-IgH-rituximab	*Rituximab H chain* gene in pMAZ-IgL	Current study
pMAZ-IgH-rituximab-YTE	*Rituximab YTE mutant H chain* gene in pMAZ-IgL	Current study
pMAZ-IgH-rituximab-LS	*Rituximab LS mutant H chain* gene in pMAZ-IgL	Current study
pMAZ-IgH-rituximab-PFc29	*Rituximab PFc29 mutant H chain* gene in pMAZ-IgL	Current study
pMAZ-IgH-rituximab-PFc29-LALA	*Rituximab PFc29-L234A/L235A mutant H chain* gene in pMAZ-IgL	Current study
pMAZ-IgH-rituximab-PFc29-LALAPG	*Rituximab PFc29-L234A/L235A/P329G mutant H chain* gene in pMAZ-IgL	Current study
pMAZ-IgH-rituximab-PFc29-TL	*Rituximab PFc29-T299L mutant H chain* gene in pMAZ-IgL	Current study
pMAZ-IgH-rituximab-PFc29-LALATL	*Rituximab PFc29-L234A/L235A/T299L mutant H chain* gene in pMAZ-IgL	Current study
pMAZ-aflibercept	*Aflibercept wild-type* gene in pMAZ-IgL	Current study
pMAZ-aflibercept-YTE	*Aflibercept YTE mutant* gene in pMAZ-IgL	Current study

Supplementary Table 2. Primers used in this study.

Primer name	Primer nucleotide sequence (5′→3′)
SHK#1	CCAGGCTTTACACTTTATGC
SHK#2	CTGCCCATGTTGACGATTG
SHK#3	CCAGCCGGCCATGGCG
SHK#4	GAATTCGGCCCCCGAGGCCCC
SHK#13	GTCCTTGGGTTTTGGGGGGAAG
SHK#14	CTTCCCCCCAAAACCCAAGGACNNKCTCATGATCTCCCGGACCCCTGA GGTCACATGCG
SHK#15	CTTCCCCCCAAAACCCAAGGACACCNNKATGATCTCCCGGACCCCTGA GGTCACATGCG
SHK#16	CTTCCCCCCAAAACCCAAGGACACCCTCNNKATCTCCCGGACCCCTGA GGTCACATGCG
SHK#17	CTTCCCCCCAAAACCCAAGGACACCCTCATGNNKTCCCGGACCCCTGA GGTCACATGCG
SHK#18	CTTCCCCCCAAAACCCAAGGACACCCTCATGATCNNKCGGACCCCTGA GGTCACATGCG
SHK#19	CTTCCCCCCAAAACCCAAGGACACCCTCATGATCTCCNNKACCCCTGA GGTCACATGCG
SHK#20	GACGGTGAGGACGCTGACC
SHK#21	GGTCAGCGTCCTCACCGTCNNKCACCAGGACTGGCTGAATGGCAAGG AGTACAAGTGCAAGG
SHK#22	GGTCAGCGTCCTCACCGTCCTGCACNNKGACTGGCTGAATGGCAAGG AGTACAAGTGCAAGG
SHK#23	GGTCAGCGTCCTCACCGTCCTGCACCAGGACTGGNNKAATGGCAAGG AGTACAAGTGCAAGG
SHK#24	CACGGAGCATGAGAAGACGTTCC
SHK#25	GGAACGTCTTCTCATGCTCCGTGCTGCATNNKGCTCTGCACAACCACT ACACGCAGAAGAGCCTCTCCCTG
SHK#26	GGAACGTCTTCTCATGCTCCGTGCTGCATGAGGCTNNKCACAACCACT ACACGCAGAAGAGCCTCTCCCTG
SHK#27	GGAACGTCTTCTCATGCTCCGTGCTGCATGAGGCTCTGCACNNKCACT ACACGCAGAAGAGCCTCTCCCTG
SHK#28	GGAACGTCTTCTCATGCTCCGTGCTGCATGAGGCTCTGCACAACCACN NKACGCAGAAGAGCCTCTCCCTG
SHK#29	CAATTTCCTCATTTTATTAGGAAAGGACAGTGGG
SHK#30	GCTGTATCATCCTCTTCTTGGTAGCAAC
SHK#31	ACAAGATTTAGGGGGCTCAACTTTCTTGTC
SHK#32	GACAAGAAAGTTGAGCCCCCTAAATCTTGTGACAAAACTCACACATGC CCACCG
SHK#33	GGGCCCTCTAGATCATTTACCCGGGGACAGGGAGAGGCT

SHK#34	CCACAGGCGCGCACTCCATCCAGAGGACCCCCAAGATCCAG
SHK#35	CGAGCTTCTAGATCACATGTCCCTGTCCCACTTCACGG
SHK#36	CCACAGGCGCGCACTCC GCCGAGAGCCACCTTAGCC
SHK#37	CGAGCTTCTAGATTATCAATGATGATGGTGGTGATGGCTGCC
SHK#38	CCACAG GCGCGC ACTCCGAAGCTATCCAGCGTACTCCAAAGATTCAG
SHK#39	CGAGCTTCTAGATCACATGTCTCGATCCCACTTAACTATCTTGGG
SHK#40	CCACAGGCGCGCACTCCGCAGAAAGCCACCTCTCCCTCC
SHK#41	CGAGCTTCTAGATTATCAATGATGATGGTGGTGATGGCTGCCCAGCTC CACCCTGAGGGGC
SHK#42	CGCAGCGAGCGCGCACTCCGAAATGGCGGAAGCTATCCAGCGTACTC CAAAGATTCAGGTTTAC
SHK#43	CTCCACTACCTCCACCCCCTGATCCGCCTCCGCCCATGTCTCGATCCCA CTTAACTATCTTGGG
SHK#44	GATCAGGGGGTGGAGGTAGTGGAGGGGGAGGTTCCGCAGAAAGCC ACCTCTCCCTCC
SHK#45	CCTTGCTATCTTTAGACGGGTCAGATCCGCTGCCCAGCTCCACCC
SHK#46	GGGTGGAGCTGGGCAGCGGATCTGACCCGTCTAAAGATAGCAAGG
SHK#47	CCCTAAAATCTAGATCATTAGTGGTGATGATGGTGGTGAGAG
SHK#48	CCACAG GCGCGCACTCCCAGATCGTCCTGAGTCAGAGCCC
SHK#49	CTTCAACCGGGGCGAGTGCTGATCTAGAAGCTCG
SHK#50	CCACAG GCGCGCACTCCCAGGTCCAGCTCCAACAGCC
SHK#51	CTTTTCATGCACCCTCACAAAGGTGG
SHK#52	CCACCTTTGTGAGGGTGCATGAAAAGGACAAAACTCACACATGCCCA CCG

Supplementary Table 3. Equilibrium dissociation constants (K_D) and rate constants (k_{on} and k_{off}) for binding between trastuzumab-Fc variants and hFcRn at pH 6.0, and RU_{max} values of trastuzumab-Fc variants upon binding to hFcRn at pH 7.4, which were determined by an SPR analysis.

	**pH 6.0**			**pH 7.4**
	**k_{on} (1/Ms)**	**k_{off} (1/s)**	**K_D (nM)**	**RU_{max}**
Trastuzumab	Steady state		646.8	1.1
Trastuzumab-YTE	1.513×10^5	2.223×10^{-2}	147	16.2
Trastuzumab-LS	2.436×10^5	1.988×10^{-2}	81.6	32
Trastuzumab-PFc29	2.027×10^5	3.013×10^{-2}	148.6	10.1
Trastuzumab-PFc41	1.517×10^5	3.005×10^{-2}	198.1	7.6

Supplementary Table 4. ADCC analysis of trastuzumab variants and CDC analysis of rituximab variants.

Trastuzumab- or rituximab-Fc variants	ADCC (Trastuzumab)		CDC (Rituximab)	
	EC_{50} (μg/ml)	Fold change relative to trastuzumab	EC_{50} (μg/ml)	Fold change relative to rituximab
Wild-type Fc	0.12	1	0.33	1
YTE	-	-	-	-
LS	0.061	1.9	0.26	1.2
PFc29	0.051	2.6	0.092	3.6

SI References

1. Andersen, J. T. et al. Ligand binding and antigenic properties of a human neonatal Fc receptor with mutation of two unpaired cysteine residues. *FEBS J.***275**, 4097-4110 (2008).
2. Jung, S. T. et al. Aglycosylated IgG variants expressed in bacteria that selectively bind FcgammaRI potentiate tumor cell killing by monocyte-dendritic cells. *Proc. Natl. Acad. Sci. U. S. A.* **107**, 604-609 (2010).
3. Jo, M., Hwang, B., Yoon, H. W. & Jung, S. T. Escherichia coli inner membrane display system for high-throughput screening of dimeric proteins. *Biotechnol. Bioeng.* **115**, 2849-2858 (2018).
4. Berntzen, G. et al. Prolonged and increased expression of soluble Fc receptors, IgG and a TCR-Ig fusion protein by transiently transfected adherent 293E cells. *J. Immunol. Methods* **298**, 93-104 (2005).
5. Fromant, M., Blanquet, S. & Plateau, P. Direct random mutagenesis of gene-sized DNA fragments using polymerase chain reaction. *Anal. Biochem.* **224**, 347-353 (1995).
6. Waterhouse, A. et al. SWISS-MODEL: homology modelling of protein structures and complexes. *Nucleic Acids Res.* **46**, W296-w303 (2018).
7. Ahmed, A. A. et al. Structural characterization of anti-inflammatory immunoglobulin G Fc proteins. *J. Mol. Biol.* **426**, 3166-3179 (2014).
8. Anandakrishnan, R., Aguilar, B. & Onufriev, A. V. H++ 3.0: automating pK prediction and the preparation of biomolecular structures for atomistic molecular modeling and simulations. *Nucleic Acids Res.* **40**, W537-W541 (2012).
9. Lauber, M. A. et al. Rapid Preparation of Released N-Glycans for HILIC Analysis Using a Labeling Reagent that Facilitates Sensitive Fluorescence and ESI-MS Detection. *Anal. Chem.* **87**, 5401-5409 (2015).
10. Cisco, R. M. et al. Induction of human dendritic cell maturation using transfection with RNA encoding a dominant positive toll-like receptor 4. *J.Immunol.* **172**, 7162-7168 (2004).
11. Shields, R. L. et al. High resolution mapping of the binding site on human IgG1 for Fc gamma RI, Fc gamma RII, Fc gamma RIII, and FcRn and design of IgG1 variants with improved binding to the Fc gamma R. *J. Biol. Chem.* **276**, 6591-6604 (2001).
